# QTL mapping and validation of fertility restoration in West African sorghum A_1_ cytoplasm and identification of a potential causative mutation for *Rf*_2_

**DOI:** 10.1007/s00122-018-3161-z

**Published:** 2018-08-21

**Authors:** Moctar Kante, Henry Frederick W. Rattunde, Baloua Nébié, Eva Weltzien, Bettina I. G. Haussmann, Willmar L. Leiser

**Affiliations:** 1 Institute of Plant Breeding, Seed Science and Population Genetics, University of Hohenheim, Fruwirthstr. 21, 70599 Stuttgart, Germany; 2 University of Wisconsin–Madison, Madison, WI USA; 3 International Crops Research Institute for the Semi-Arid Tropics, BP 320, Bamako, Mali; 4 State Plant Breeding Institute, University of Hohenheim, Fruwirthstr. 21, 70599 Stuttgart, Germany

## Abstract

**Abstract:**

***Key message*** **Major A**_**1**_ **cytoplasm fertility restoration loci,** ***Rf***_**2**_ **and** ***Rf***_**5**_ **, were found in the West African sorghum. A potential causative mutation for** ***Rf***_**2**_ **was identified. KASP markers were validated on independent material.**

**Abstract:**

To accelerate the identification and development of hybrid parental lines in West African (WA) sorghum, this study aimed to understand the genetics underlying the fertility restoration (*Rf*) in WA A_1_ cytoplasmic male sterility system and to develop markers for a routine use in WA breeding programs. We genotyped by sequencing three F_2_ populations to map the *Rf* quantitative trait loci (QTL), validated the molecular KASP markers developed from those QTL in two F_2:3_ populations, and assessed the most promising markers on a set of 95 R- and B-lines from WA breeding programs. Seven QTL were found across the three F_2_ populations. On chromosome SBI-05, we found a major fertility restorer locus (*Rf*_5_) for two populations with the same male parent, explaining 19 and 14% of the phenotypic variation in either population. Minor QTL were detected in these two populations on chromosomes SBI-02, SBI-03, SBI-04 and SBI-10. In the third population, we identified one major fertility restorer locus on chromosome SBI-02, *Rf*_2_, explaining 31% of the phenotypic variation. Pentatricopeptide repeat genes in the *Rf*_2_ QTL region were sequenced, and we detected in Sobic.002G057050 a missense mutation in the first exon, explaining 81% of the phenotypic variation in a F_2:3_ population and clearly separating B- from R-lines. The KASP marker developed from this mutation stands as a promising tool for routine use in WA breeding programs.

**Electronic supplementary material:**

The online version of this article (10.1007/s00122-018-3161-z) contains supplementary material, which is available to authorized users.

## Introduction

*Sorghum bicolor* (L.) Moench is of vital importance for many smallholder farmers and their families in various regions of West Africa (WA). However, average grain yields for farmers in this zone are of 1 t/ha. Sorghum hybrids for WA were explored between the 1960s and 1990s (Andrews 1975; Toure and Scheuring 1982; Atokple 2003), but no hybrids were released or commercialized. A major challenge with the initial hybrids was their poor and unacceptable grain quality (Toure and Scheuring 1982). Recent work in WA to develop both male and female parents using the widely cultivated sorghum Guinea-race germplasm has resulted in hybrids with suitable grain quality combined with yield superiorities over farmer’s local varieties under diverse productivity conditions (Rattunde et al. 2013; Kante et al. 2017; Weltzien et al. 2018). The positive results with these hybrids under on-farm farmer-managed testing, and indications that farmers are starting to adopt these new hybrids (Smale et al. 2014) justify establishing a full-scale hybrid breeding pipeline to produce hybrids that meet farmers’ demands in this major sorghum producing zone.

Intensive hybrid breeding and seed production in many crop species were made possible by the identification and characterization of a stable and heritable cytoplasmic male sterility (CMS) mechanism. CMS is a maternally inherited defect where, as the result of specific nuclear and mitochondrial interactions, plants fail to produce functional pollen, or to ensure normal anther dehiscence, without affecting the female fertility (Duvick 1959; Laughnan and Gabay-Laughnan 1983; Hanson and Conde 1985; Levings and Brown 1989). It is attributed to abnormal transcripts usually coding for chimeric open reading frames (ORFs) (Schnable and Wise 1998; Tang et al. 1998; Hanson and Bentolila 2004). A CMS system depends therefore on a set of male-sterility-causing cytoplasms and dominant or recessive alleles in the nuclear genome, which either restore the fertility or maintain the sterility (Maunder and Pickett 1959; Rooney and Wayne Smith 2000). Several male-sterility-inducing cytoplasms were described in sorghum since the identification of a stable CMS system by Stephens and Holland (1954).

The A_1_ (*milo*) cytoplasm was first documented (Conner and Karper 1927) and is most widely used in sorghum hybrid development worldwide, followed by the A_2_ cytoplasm (Schertz 1977; Schertz and Ritchey 1978). Other types of CMS, namely A_3_ (Quinby 1980), A_4_ (Rao et al. 1984; Worstell et al. 1984), A_5_, A_6_, 9E (Webster and Singh 1964) and KS (Ross and Hackerott 1972), were also described. However, their use in commercial hybrid breeding programs is limited by the negative effects on grain yield of A_3_ cytoplasm (Moran and Rooney 2003), the low environmental stability of the restoration and, consequently, the lack of elite male restorer lines.

Fertility is restored in the progenies when a cytoplasmic male-sterile female is crossed with a male carrying the corresponding nuclear-encoded genes (restorer of fertility, *Rf*) that partially or completely restore the fertility. Recent quantitative trait loci (QTL) studies that used American, Australian and Indian sorghum material found, depending on the restorer line and the CMS type involved, one or several major loci controlling the fertility restoration in the A_1_ and A_2_ cytoplasms, complemented by genes with smaller effects (partial restorer) and modifier genes (Maunder and Pickett 1959; Murty and Gangadhar 1990; Qian 1990; Klein et al. 2001, 2005; Wen et al. 2002; Sanjana Reddy et al. 2010; Jordan et al. 2011; Yin et al. 2013). The so-far-suggested sorghum *Rf* genes for the sorghum A_1_ and A_2_ cytoplasm encode proteins that belong to the pentatricopeptide repeat (PPR) protein family (Klein et al. 2005; Jordan et al. 2010, 2011).

The A_1_-cytoplasm *Rf*_1_ locus was mapped on linkage group (LG) 8 (Klein et al. 2001), which corresponds to sorghum chromosome SBI-08 (Peng et al. 1999; Kim et al. 2005a, b). Two microsatellite markers flanking the *Rf*_1_ locus covered a 22.8-cM region with low recombination frequencies of 6.5 and 6.8% between the two markers and the gene. Further, sorghum *PPR13* was cloned and reported as a candidate for the sorghum *Rf*_1_ gene (Klein et al. 2005). *Rf*_2_, a major A_1_-cytoplasm fertility restorer locus mapped on chromosome SBI-02, explained approximately 87% of the seed-set phenotypic variation (Jordan et al. 2010). The presence of modifier genes, revealed by a continuum of partially restored fertility, was observed in both used populations. Further, the locus on chromosome SBI-02 includes one PPR gene (Sb02g004810.1 with Sbi1.4 reference genome and Sobic.002G057050 with the Sbi3.4 reference genome) that co-segregates with the fertility restoration phenotype and could be a potential candidate gene. Recently, the *Rf*_5_ locus, located on chromosome SBI-05, has been associated with the fertility restoration in both A_1_ and A_2_ cytoplasms (Jordan et al. 2011). It explained 42 and 45% of the phenotypic variation in percent seed set on the A_1_ and A_2_ cytoplasm, respectively. A minor or partial restorer locus was also detected on chromosome SBI-04 explaining 10% of the variation of the percent seed set. Moreover, the *Rf*_6_ fertility restoration locus was mapped on chromosome SBI-04 and restores fertility in both A_1_ and A_2_ cytoplasms (Praveen et al. 2015). Informative markers based on these *Rf* genes could help in a cost-effective marker-assisted screening of breeding material for developing and identifying fertility restorer or maintainer lines.

A good understanding of the restoration and maintenance capacity of WA germplasm is essential for a long-term WA hybrid breeding program using the A_1_-type cytoplasm. To date, however, a detailed genetic study of fertility restoration in West African and Guinea-race germplasm has not been done, with prior studies focusing on Australian, Indian and US breeding materials. The Guinea-race of sorghum, predominantly cultivated across WA from Senegal to the western border of Nigeria, is known to be most genetically diverse and distinct from the other major sorghum races (Folkertsma et al. 2005; Deu et al. 2006). Most of the WA sorghum germplasm restores the A_1_ cytoplasm, and therefore, it is essential to have tools in hand which would ease the identification of potential maintainer and restorer lines.

In order to accelerate the development of hybrid parental lines for WA and facilitate broadening the genetic pool of WA sorghum females, this study aims to understand the underlying genetics of pollen fertility restoration in key WA hybrid parents, to develop diagnostic and cost-efficient molecular markers for fertility restoration that can be used in applied WA hybrid breeding programs, and to assess the potential utility of those markers. We report here (1) the evaluation of several pollen fertility restoration traits and relationships among them, (2) the identification of QTL for fertility restoration in the A_1_ CMS system in WA germplasm and (3) molecular markers developed for further use in WA sorghum hybrid breeding programs.

## Materials and methods

### F_2_-based mapping study

#### Plant material

Three F_2_ populations derived from the hybridization of two A- and two R-lines were created for the segregation analysis. The two female inbred lines, “Combine Kafir-60” [CK60A] and FambeA, have the A_1_-type of CMS. FambeA and the two male inbred lines, 97-SB-F5DT-298 [hereafter called DT_298] and Lata, are widely used parents in hybrid breeding research in Mali. FambeA is derived from a Malian Guinea-race local variety, Lata was derived from a random-mating population based mostly on Guinea landrace germplasm but with somewhat more than 12% introduced genetic background, while DT_298 was the product of bi-parental pedigree breeding with inter-racial Guinea-Caudatum parentage. CK60A is an old A-line developed in Texas (USA), which is known to have fewer genes leading to partial fertility, and was therefore used as the source of sterile cytoplasm in creating FambeA.

The three F_1_ crosses, CK60AxDT_298 [POP_CD_, Caudatum × inter-racial intercross], FambeAxDT_298 [POP_FD_, Guinea race × inter-racial intercross] and FambeAxLata [POP_FL_, Guinea × Guinea intercross] were developed in the 2014 rainy season, at the International Crops Research Institute for the Semi-Arid Tropics (ICRISAT) research station, near Bamako, Mali. Randomly chosen F_1_ plants were selfed in the 2015 off-season, and F_2_ seeds of one F_1_ plant of each of the three crosses were harvested, hand-threshed and stored in a cold chamber.

#### Field trial and phenotyping

For each F_2_ family, 220 hills were sown on June 8, 2015, in two bands of 10 ridges each. The 3-m long ridges were separated by 75 cm with 11 hills per ridge. The parental male lines were sown in two rows and each A-line in three rows. Two weeks after sowing, each hill was thinned to one plant. The trial was weeded 3 weeks after sowing, and the operation was repeated frequently throughout plant development. Basic soil fertilization was applied with 100 kg ha^−1^ of diammonium phosphate while preparing the soil before sowing. Organic manure was hand-applied directly in the hill-holes prior to sowing, and 50 kg ha^−1^ of urea was applied after the first weeding. Due to low vigor and mortality in the field, only 168, 125 and 175 F_2_ plants were phenotyped in POP_CD_, POP_FD_ and POP_FL_, respectively (Table 1). All available F_2_ panicles and three random panicles of each parental line were selfed at heading stage with paper bags. The bags were removed only for harvest to avoid cross-pollination and bird damage. Daily temperatures during the flowering period (from July 30 to October 11, 2015) were within the normal range of sorghum production temperatures in Mali (18 °C min. in the mornings and 45 °C max. in the midday–early afternoon). A direct impact of high temperatures on panicle fertility was not observed in any of our trials.

**Table 1:** Details of the sorghum F_2_ populations used in the mapping study

Population	Cross	Racial background^a^	Number of genotyped individuals	Number of individuals used for mapping
POP_CD_	CK60AxDT-298	CxCG	168	166
POP_FD_	FambeAxDT-298	GxCG	125	124
POP_FL_	FambeAxLata	GxG	175	174

^a^C, CG and G denote Caudatum, Caudatum-Guinea inter-racial and Guinea, respectively

F_2_ panicles were individually harvested at maturity and visually evaluated for seed set using a fertility restoration score. The fertility score used a 0–10 scale with 0 for complete sterility with no seed set and 10 for fully fertile panicles with complete seed set. The classification of panicles for fertility phenotype considered panicles with scores of 0–2 to be sterile (i.e., maintainer reaction) and those with fertility scores of 8–10 to be fertile (i.e., fertility restorer reaction), with all remaining intermediate fertility scores as partially fertile. The Chi-square (*χ*^2^) testing segregation ratios for major fertility genes used fertility score ranges of 0–2 as sterile and 3–10 as fertile. Besides, panicle length, panicle dry weight, threshed grain weight and 100 seed weight were directly observed and grain number per panicle was estimated using grain weight and 100 seed weight. Due to the ambiguous fertility score distributions in POP_CD_ and POP_FD_, we set up a validation study during the 2016 rainy season using 100 F_2_ plants from the remaining seeds of each of the F_2_ populations. Fertility score was recorded in these repeated trials as in the F_2_ mapping populations.

#### Genotyping

*Leaf sampling and DNA extraction* Three weeks after sowing, when plants were at stage 2 (Vanderlip and Reeves 1972), leaf samples were collected from each viable F_2_ plant. With a leaf puncher, 30 disks per plant were put in a labeled tea bag and directly dried on silica gel.

DNA extraction and purification followed the cetyltrimethylammonium bromide (CTAB) protocol with 20 disks of leaf sample per individual F_2_ plant. The extracted DNA was solubilized into 100 µL of TE buffer. Finally, the 482 samples were diluted to 30–100 ng/µL and shipped to Cornell University for genotyping by sequencing (GBS) (Elshire et al. 2011). These samples comprised the 168, 125 and 175 individuals from POP_CD_, POP_FD_ and POP_FL_ (Table 1), along with two samples of the female CK60A and four of each of the three remaining parents, FambeA, Lata and DT_298.

*Sequence analyses and single nucleotide polymorphism (SNP) calling and filtering* GBS libraries were constructed in 192- and 96-plex using the ApeKI restriction enzyme. The TASSEL 5 (Trait Analysis by Association, Evolution and Linkage) GBS v2 pipeline (Glaubitz et al. 2014) was used to extract informative SNPs from the raw sequencing data. Alignment of tags to the *Sorghum bicolor* reference genome version 3 (McCormick et al. 2018) was achieved using the Burrows–Wheeler alignment (BWA) tool (Li and Durbin 2009). Finally, we obtained with no specific SNP filtering 148,376 SNPs for all three F_2_ populations and parental lines.

With TASSEL 5, a cladogram was constructed with parental and F_2_ individuals to confirm the supposed crosses made in the field. Four F_2_ individuals that were detected as outliers were removed from the study (Table 1).

Using R (R Development Core Team 2011) and VCFtools (Danecek et al. 2011), individual populations were filtered by removing monomorphic sites between both parents, sites containing missing data for any of the parents, as well as sites with a coverage < 10 and/or a minor allele frequency (MAF) < 0.2. After filtering, 7821, 5701 and 5197 sites remained in POP_CD_, POP_FD_ and POP_FL_, respectively. Missing data were then imputed with *FSFHap* in TASSEL 5. POP_CD_, POP_FD_ and POP_FL_ had 6.6, 2.8 and 11.9% of missing data before any imputation and 6.1, 2.5 and 11.4% of missing data after the TASSEL imputation. Due to undercalling of heterozygous loci using low coverage GBS data, we corrected the imputed data with ABHGenotypeR package (Reuscher and Furuta 2016). Undercalled heterozygous and short miscalled stretches were corrected based on flanking alleles with a window (*maxHapLength*) of four markers. This procedure reduced missing data to 2.0, 0.7 and 3.7% in POP_CD_, POP_FD_ and POP_FL_, respectively.

#### Linkage map construction and QTL mapping

Prior to the map construction, *χ*^2^-tests were conducted for all SNPs of all populations to detect segregation-distorted sites. Distorted markers (*p* < 0.001; 44, 12 and 65% of markers for POP_CD_, POP_FD_ and POP_FL_, respectively), as well as duplicated sites, were removed with R/qtl (Broman et al. 2003). After this filtering step, the remaining 3859, 4119 and 1574 SNPs for POP_CD_, POP_FD_ and POP_FL_, respectively, were used for the linkage map construction. The R/qtl cross-file was converted into a BC_0_F_2_ format with the *convert*2*bcsft* function for a proper mapping input-data format for the package ASMap (Wu et al. 2008; Taylor and Butler 2017). The Kosambi mapping function, implemented with the *MSTmap* algorithm, was used for the linkage map construction, with a *p* value of 1 × 10^−6^, and for imputing the remaining missing marker data. Marker order was controlled based on the physical positions, and individual chromosome orientation was flipped when required. A composite interval mapping (CIM) analysis with the fertility score was executed using R/qtl, and the QTL allele probability was computed for every ten centimorgan with five background markers. The LOD significance was assessed following the method described in Van Ooijen (1999), and the Bayesian confidence interval, as well as the phenotypic variance explained by significant QTL, was calculated. A 10,000-permutation test was computed on all populations, separately, to estimate the genome-wide significance threshold. A 200 independently sampled fivefold cross-validation was computed using Plabmqtl (Utz 2012) with the dominance and two-loci additive × additive epistatic effects included into the model. These analyses provided information on the robustness on the detected QTL.

### KASP marker development

The SNP markers detected within and flanking the QTL regions on chromosome SBI-05 and SBI-02 were converted into kompetitive allele-specific polymerase chain reaction (KASP) markers. DNA sequences flanking the respective SNP, primer sequences and polymerase chain reaction (PCR) conditions are shown in Supplemental Table S1. All KASP assays were established and run on the Roche LightCycler480II using 10 µL PCR volumes and the KASP master mix with low ROX provided by LGC Genomics (www.lgcgroup.com).

#### Candidate gene sequencing

Pentatricopeptide repeat (PPR) genes are known to be involved in fertility reactions; hence, we Sanger-sequenced a set of selected PPR genes in the QTL regions on chromosome SBI-05 for POP_CD_ and POP_FD_. Due to the narrow confidence interval on chromosome SBI-02 (1.2 cM) and no known PPR genes within the confidence interval, we selected some of the closest PPR genes upstream of the QTL interval on chromosome SBI-02 and Sanger-sequenced them. The reference sequences and the functional annotations of the selected genes were retrieved from Phytozome (Goodstein et al. 2012, www.phytozome.com, Table 2). The primer sequences and the used PCR conditions are shown in Supplemental Table S2. We first sequenced the four parents. If we detected any potential mutations (missense, frameshifts, etc.), which discriminated the female and male parents, we used a set of other diverse B- and R-lines from ICRISAT breeding program (Supplemental Table S3) and sequenced them for the region of interest. Only in Sobic.002G057050, we detected a missense mutation, which could be validated to differentiate well B- and R-lines. This SNP was finally converted into a KASP marker named S002G057050_1090.

**Table 2:** Pentatricopeptide repeat (PPR) genes selected as candidate genes for pollen fertility reaction in WA sorghum and used for sequencing and marker creation in three F_2_ sorghum populations, their start end ending points in base pair and their functional annotations retrieved from www.phytozome.com

Population	Gene	Start	End	Gene length (bp)	Functional annotation
POP_CD_ + POP_FD_	Sobic.005G011000	982,653	985,317	2664	Similar to *Rf*_1_ protein, mitochondrial, putative, expressed
POP_CD_ + POP_FD_	Sobic.005G017100	1,521,737	1,525,057	3320	Similar to Pentatricopeptide, putative, expressed
POP_CD_ + POP_FD_	Sobic.005G020600	1,910,905	1,915,075	4170	Similar to Os11g0128700 protein^a^
POP_CD_ + POP_FD_	Sobic.005G026400	2,368,144	2,371,917	3773	(1 of 437) PF13041—PPR repeat family (PPR_2)
POP_FL_	Sobic.002G054100	5,155,027	5,157,017	1190	(1 of 18) PF12854//PF13041—PPR repeat (PPR_1)//PPR repeat family (PPR_2)
POP_FL_	Sobic.002G057050	5,546,273	5,550,944	4671	(1 of 30) PF01535//PF12854//PF13041—PPR repeat (PPR)//PPR repeat (PPR_1)//PPR repeat family (PPR_2)
POP_FL_	Sobic.002G059700	5,733,343	5,735,193	1580	(1 of 30) PF01535//PF12854//PF13041—PPR repeat (PPR)//PPR repeat (PPR_1)//PPR repeat family (PPR_2)

^a^Non-PPR Sobic.005G020600 was also tested due to its similarity with Os11g0128700, which is highly expressed in rice inflorescence (www.ncbi.nlm.nih.gov)

All of the 11 KASP markers shown in Supplemental Table S1 were used in the validation study for different purposes. First, with one of the KASPs, namely S2_6843380 on chromosome SBI-02, we re-genotyped the entire POP_FL_ F_2_ family to validate the accuracy of our GBS derived SNPs and our imputation steps. A very high match (93%) between raw GBS data and genotyped F_2_ individuals, and a 99% agreement with the imputed data indicated few genotyping errors in our markers. Secondly, we used the KASPs on chromosome SBI-05 and the KASPs on chromosome SBI-02 to be validated in F_2:3_ plants of POP_CD_ and POP_FL_, respectively.

### QTL validation in F_3_ families

A single F_2_ panicle from both POP_CD_ and POP_FL_ was selected based on the high number of seeds and high proportion of heterozygous SNPs. The 92 POP_CD_ and 93 POP_FL_ F_2:3_ seeds from these panicles were used for validation of detected QTL. In the 2016 rainy season, all seeds were sown at the ICRISAT station, Samanko, with the same agronomic treatments as in the F_2_ populations in 2015.

Panicles were covered with selfing bags at heading stage. Individual bags were temporarily removed 5–7 days later to score the pollen quantity, anther color and the presence or absence of an anther pore. Pollen quantity was scored before 10 a.m. using a visual rating from one (no pollen) to five (high pollen quantity) by gently shaking each panicle and observing the pollen quantity shed. Further, the color of the anthers was rated following a visual scoring of 1 (very bright yellow to white) to 5 (dark yellow). Panicles with a pore on the anthers were scored 1 and those without pore 0. Cross-pollination from neighboring plants was prevented by having all neighboring plants bagged during individual panicle scoring and washing the hands with ethanol if necessary before opening a new bag. At maturity, all bags were removed and panicles harvested and dried. Apart from panicle length, all data recorded in the F_2_ populations (used in the mapping study) were also evaluated in the F_3_ validation populations. Leaf samples were taken from all F_2:3_ plants, dried in silica gel and brought to the University of Hohenheim for DNA extraction and KASP marker analyses. The F_2:3_ plants from POP_CD_ (hereafter called POP_CD__F_3_) were analyzed with the KASP markers on chromosome SBI-05, whereas the F_2:3_ plants from POP_FL_ (hereafter called POP_FL__F_3_) were analyzed with the KASPs on chromosome SBI-02. The phenotypic variances explained by each marker were tested in a linear regression model with fertility score as depending variable.

### QTL validation in R- and B-lines

Additionally to the QTL validation in the F_3_ families, a validation of the most promising markers was conducted using a diverse set of 95 lines of interest for the hybrid breeding programs of ICRISAT-Mali and the Institut d’Economie Rurale (IER-Mali) (Supplemental Table S3). These lines were classified as fertility restorers (R-lines, *N* = 50) or maintainers (B-Lines, *N* = 45) based on prior classification or by testcrossing those lines onto the male-sterile female CK60A during the 2017 rainy season and evaluating the fertility of the resulting hybrids. KASPs either being the QTL peak or spanning the QTL region, namely S2_6045380, S002G057050_1090, S2_6843380, S5_1180493 and S5_2174322, were evaluated for these 95 lines.

## Results

### Descriptive analysis of fertility phenotypes in three F_2_ populations

**Fig. 1:**
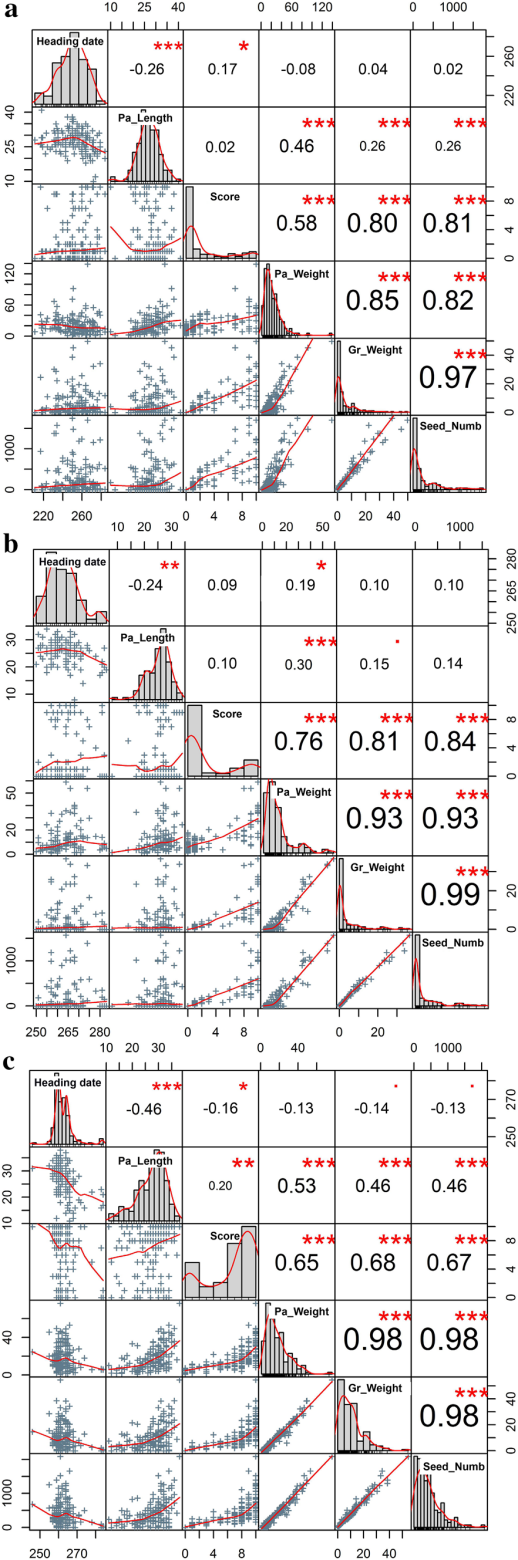
Frequency distribution of- and correlations between- phenotypic traits recorded on POP_CD_ (**a**), POP_FD_ (**b**) and POP_FL_ (**c**) sorghum F_2_ mapping populations, with 166, 124 and 174 individuals, respectively. Pa_Length, Pa_Weight and Gr_Weight are the panicle length, the panicle dry weight and the individual panicle grain weight and seed number, respectively

The fertility scores of the parents corresponded to expectations; all female parents were completely sterile, and male parents were completely fertile. The fertility scores of POP_FL_ F_2_ plants showed high frequency of fertile panicles (104 of 174 panicles) and low frequency of sterile panicles (*n* = 33), whereas POP_CD_ and POP_FD_ showed low frequency of fertile panicles (19 of 166, and 27 of 124 panicles, respectively) and a high frequency of sterile panicles (*n* = 123 and 87, respectively) (Fig. 1). Partially fertile panicles with intermediate fertility scores were observed in all populations, but their total numbers were less than that of the combined classes of fertile and sterile panicles (Fig. 1). The *χ*^2^ test revealed no statistically significant deviation from the 3:1 segregation ratio at *α* = 5% in POP_FL_ (*χ*^2^ = 3.52 with one degree of freedom). POP_CD_ and POP_FD_ deviated significantly from the 3:1 segregating ratio (*χ*^2^ = 208.29 for POP_CD_ and 137.41 for POP_FD_, with one degree of freedom).

No strong correlations were found between fertility score with measures of phenology (heading date) or panicle length (Fig. 1). The fertility score was strongly correlated with measures of panicle weight, threshed grain weight and seed number.

### Genetic maps

The linkage maps of the three F_2_ populations consisted of 10 LG (chromosome SBI-01 to SBI-10), which spanned a cumulative distance of 1602.4 cM for POP_CD_, 1736.7 cM for POP_FD_ and 1518.3 cM for POP_FL_ (Supplemental Fig. S1). The number of SNP markers in each LG varied from 66 markers on chromosome SBI-06 of POP_FL_ to 700 markers on chromosome SBI-01 of POP_FD_, with an average of 386, 412 and 157 SNPs per linkage group for POP_CD_, POP_FD_ and POP_FL_, respectively. The average distance between markers across the 10 linkage groups was 0.4 cM for both POP_CD_ and POP_FD_, and 1.0 cM for POP_FL_.

### QTL analyses

**Fig. 2:**
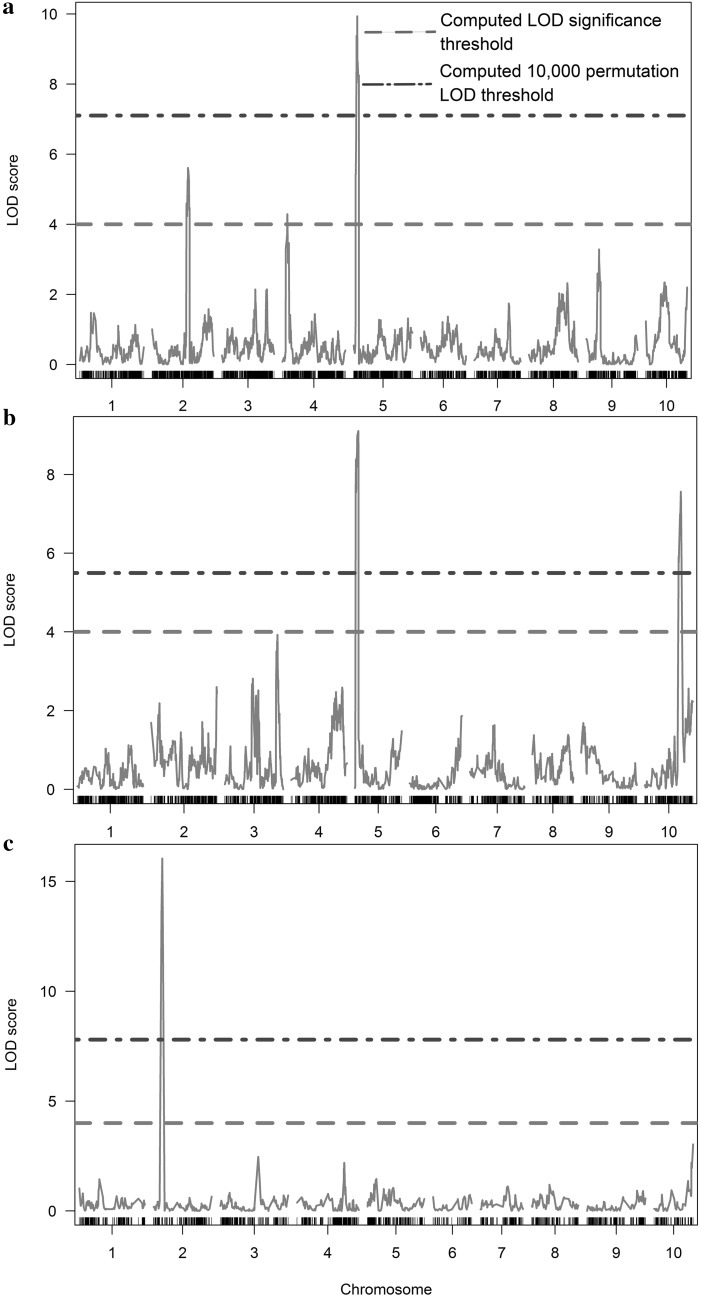
QTL scan from composite interval mapping of the fertility (A_1_ cytoplasm) score for three sorghum F_2_ populations (POP_CD_ = a; POP_FD_ = b; POP_FL_ = c)

Seven significant QTL (LOD score > 4) for fertility restoration were identified on five of the ten sorghum chromosomes based on associations with fertility scores across the three F_2_ populations (Fig. 2, Table 3). The QTL that explained the greatest phenotypic variation for fertility score were identified on SBI-02 in POP_FL_ (Table 3), accounting for over 31% and having 97% match in cross-validation runs. The next QTL, in order of percent of phenotypic variation explained, were identified on SBI-05 in POP_CD_ and POP_FD_ and on SBI-10 in POP_FD_. These QTL had LOD scores not only superior to four (computed LOD significance threshold) but also exceeding the population-specific 10,000-permutation LOD thresholds (Fig. 2, Table 3). In addition, QTL were identified on chromosome SBI-05 of POP_CD_ and POP_FD_ with 76 and 62% of matches from the cross-validation runs, respectively.

**Table 3:** QTL identified for the male fertility restoration (A_1_ cytoplasm) visual score in three sorghum F_2_ populations

Population	Number of markers	Chr	Peak SNP	Position (cM)	LOD	LOD 10,000 permutations (5%)	% Phenotypic variance explained	Bayesian confidence interval (cM)	% Match in 200 independent fivefold cross-validation runs
POP_CD_	3859	2	S2_62296150	107.8	5.6	7.1	8.6***	103.4–112.6	16
		4	S4_3253724	15.3	4.3	7.1	6.1**	10.4–19.5	33
		5	S5_1608322	10.3	9.9	7.1	18.9**	7.9–11.8	76
POP_FD_	4119	3	S3_71313485	167.8	4.3	5.5	2.5	85.1–171.4	22
		5	S5_2174322	7.7	9.6	5.5	14.3***	4.8–7.9	62
		10	S10_54552031	115.1	7.5	5.5	16.2***	112.4–116.6	37
POP_FL_	1574	2	S2_7063629	24.6	16.0	7.8	31.0***	24.0–25.2	97

**, ***Significant at 0.01 and 0.001, respectively

### Candidate gene sequences

**Fig. 3:**
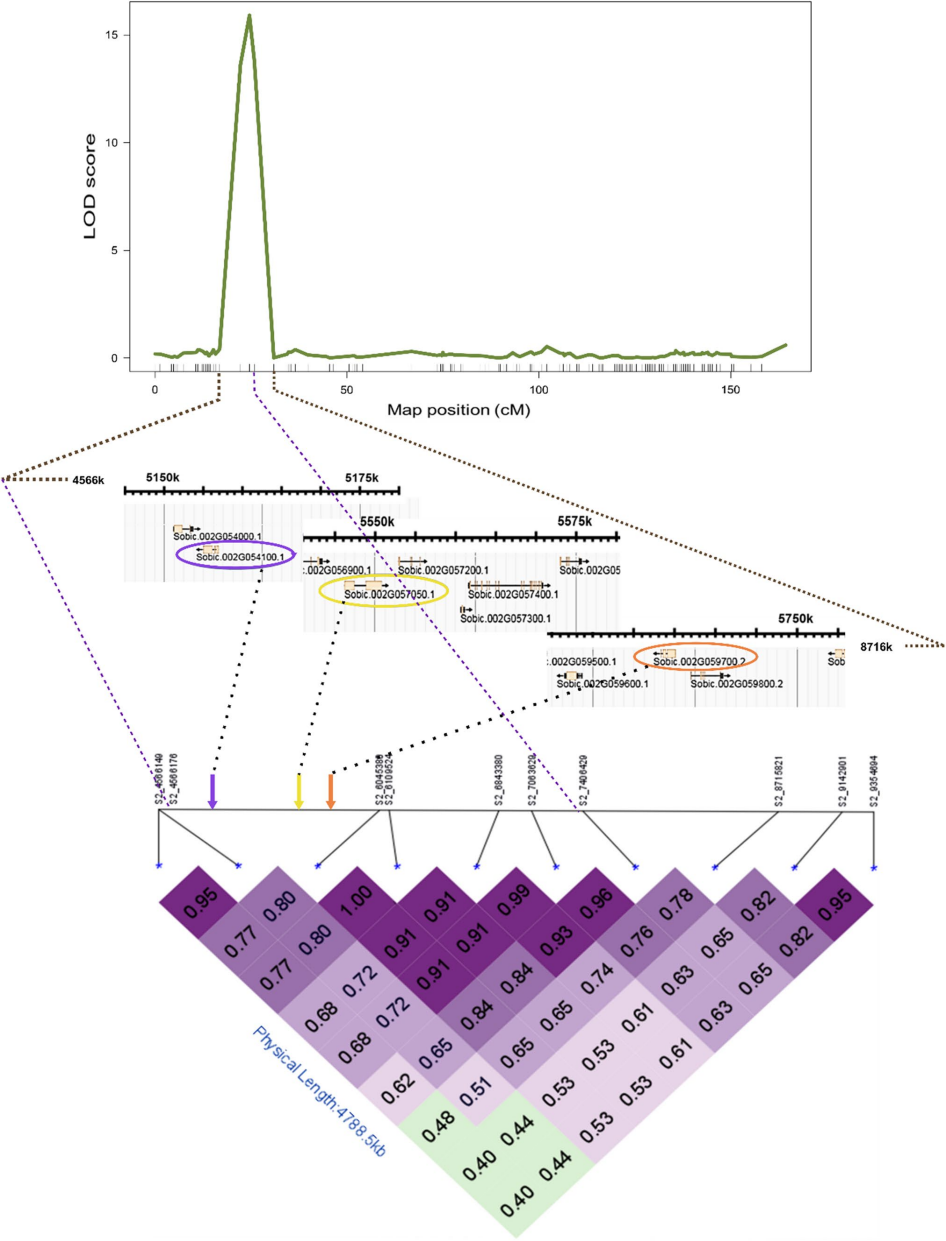
Scan of QTL for male fertility reaction (A_1_ cytoplasm) in sorghum POP_FL_ for chromosome SBI-02, and a high-resolution genome map showing the selected and sequenced PPR genes and their positions (indicated with arrows) among the SNPs within this region. The heatmap shows the *R*^2^ among the different SNPs, delimiting three major linkage blocks, whereas the QTL peak falls within the middle one

**Fig. 4:**
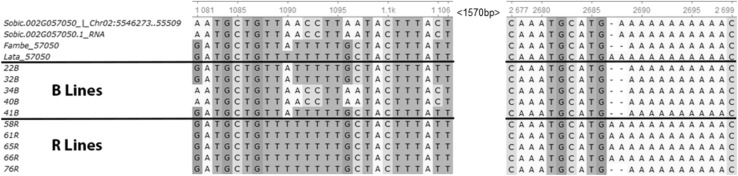
Aligned sequences of Sobic.002G057050 around the two mutations at 1090 bp and 2686 bp, putatively related to male fertility reaction in sorghum. The two first rows show the sorghum reference genome, followed by the parents of POP_FL_ mapping population, Fambe (A-line) and Lata (R-line), and followed by five B- and five R-lines sampled from ICRISAT breeding material (Supplemental Table S3)

Three candidate PPR genes on chromosome SBI-02, close to the narrow confidence interval of the detected QTL in POP_FL_ mapping population, were sequenced (Table 2, Fig. 3). Several mutations for Sobic.002G054100 were detected, but only one missense SNP (262 bp) caused an amino acid change. However, sequencing R- and B-lines for Sobic002g054100, we could not see any consistency and discriminating ability between R- and B-lines at this position. Direct evidence of effect of this gene is therefore lacking. No SNP was detected in Sobic.002G059700 between the parents of POP_FL_. Several mutations in Sobic.002G057050 were detected, including one missense SNP (1090 bp) and one 2 bp InDel (2687–2688 bp) causing a frameshift mutation. These two mutations were also detected in multiple R- and B-lines, differentiating the groups very well, except for one R-line (76R) that contained the deletion (Fig. 4, see Supplemental Table S3). The SNP at position 1090 bp was therefore chosen and converted into a KASP marker for further validation.

Sequencing candidate genes on chromosome SBI-05 (Table 2) did not result in the detection of any mutations among the parental lines for Sobic.005G011000. Several SNPs in Sobic.005G017100 were detected in the first exon, but none of these mutations clearly separated R- and B-lines. Several SNPs in the 3′-UTR were detected in Sobic.005G020600 and Sobic.005G026400, but none of them could be validated by effectively discriminating among R- and B-lines. We cannot rule out that Sobic.005G017100, Sobic.005G020600 and Sobic.005G026400 are somehow involved in the fertility reaction caused by the QTL on chromosome SBI-05 since we only sequenced a limited number of R- and B-lines.

### Validation of QTL

**Fig. 5:**
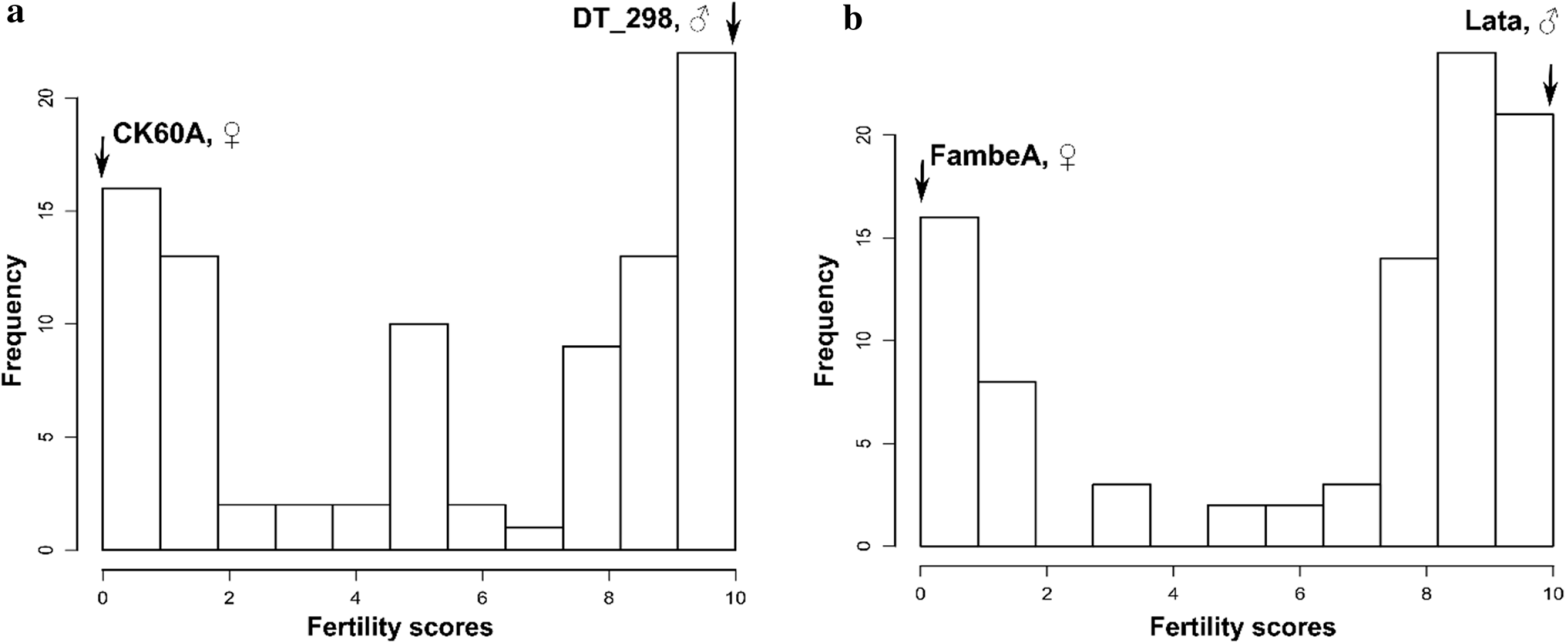
Frequency distribution of fertility (A_1_ cytoplasm) scores of 92 F_3_ plants of POP_CD__F_3_ (**a**) and 93 F_3_ plants of POP_FL__F_3_ (**b**) validation populations and parental means

The female and male parents in the validation study showed complete sterility (score = 0) and fertility (scored 10), respectively (Fig. 5). In POP_CD__F_3_, 34% of the 92 scored panicles were sterile, and 48% were fertile. In POP_FL__F_3_, 26% of the 93 scored panicles were sterile, and 63% were fertile. Both F_3_ populations showed plants with partial fertility representing 18% and 11% of the total plants in POP_CD__F_3_ and POP_FL__F_3_, respectively. The *χ*^2^ test revealed a statistically significant deviation from the 3:1 segregation ratio at α = 5% for POP_CD__F_3_ population (*χ*^2^ = 83.71 with one degree of freedom). POP_FL__F_3_ approximated the 3:1 fertile to sterile segregation ratio (*χ*^2^ = 0.03 with one degree of freedom) (Fig. 5). Panicle weight, grain weight and grain number had the same trend as the fertility-related scores.

The fertility scores of the F_3_ validation plants showed no correlation with heading date in either POP_CD__F_3_ or POP_FL__F_3_, but were positively correlated with pollen quantity (*r* = 0.57 and *r* = 0.79, respectively) and negatively correlated with anther color (*r* = − 0.60) for POP_FL_. The fertility scores of both F_3_ populations were strongly correlated with panicle weight, grain weight and seed number (ranging from 0.64 to 0.68 for POP_CD__F_3_ and 0.70 to 0.78 for POP_FL__F_3_; *α* < 0.001) similarly to the F_2_ populations.

**Fig. 6:**
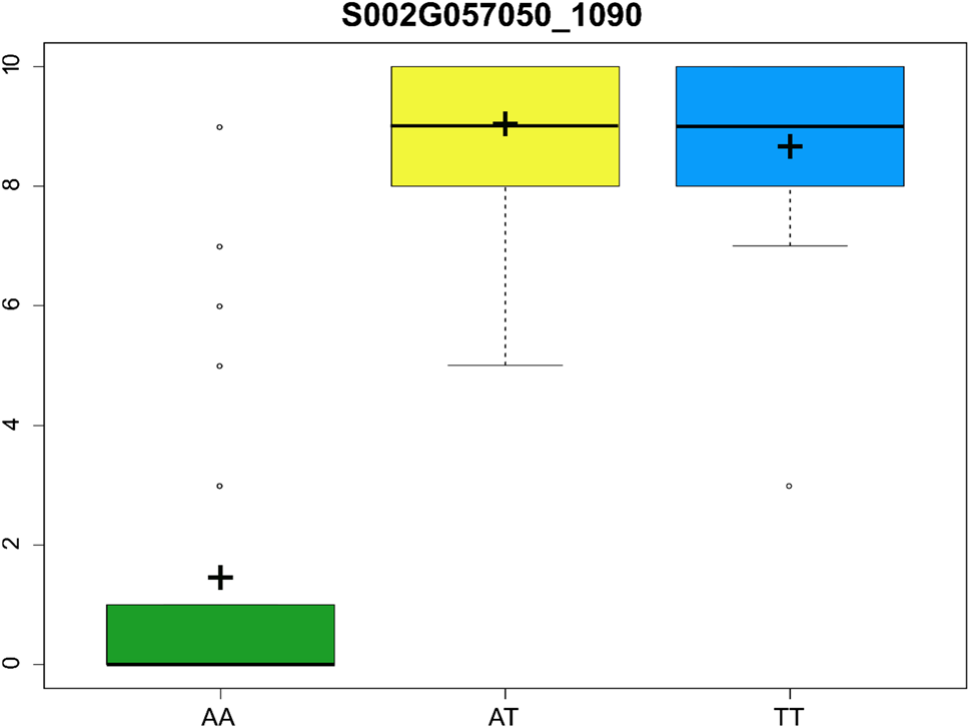
Boxplot showing the fertility scores of different F_3_ individuals as related to their genotypic values for the KASP marker Sobic.002G057050_1090. *A* is the recessive (female/maintainer) allele, and *T* is the dominant (restorer) allele. Crosses indicate the mean and the horizontal line the median of each group

Markers on chromosome SBI-05 explained between 0.97 and 7.83% of the phenotypic variation of POP_CD__F_3_, thus showing a very low consistency between the mapping and validation study (Table 4). Contrarily, the markers on chromosome SBI-02 explained between 54 and 81% of the variation in POP_FL__F_3_. KASP S002G057050_1090, developed from the missense SNP in Sobic.002G057050 (1090 bp), explained 81% of the variation in POP_FL__F_3_ (Table 4, Fig. 6). However, it explained only 25% of the phenotypic variation in the F_2_ mapping population (data not shown). The KASP S2_6843380, co-located with our peak SNP in the linkage map, explained 62% and 30% of the phenotypic variation in the POP_FL__F_3_ validation set and in the F_2_ mapping population, respectively.

**Table 4:** Percent of phenotypic variance for fertility (A_1_ cytoplasm) score explained by the newly developed KASP markers in POP_CD__F3 and POP_FL__F3 sorghum F_3_ validation populations

Population	Markers	% Variance explained in F_3_ populations
POP_CD__F_3_	S5_1180493	1.0
	S5_1608322	1.5
	S5_1610046	2.0
	S5_2174322	1.6
	S5_254752	1.9
	S5_3626674	7.8*
POP_FL__F_3_	S002G057050_1090	80.9***
	S2_6045380	77.5***
	S2_6843380	62.7***
	S2_7406429	54.1***

*, ***Significant at *α* = 0.05 and 0.001, respectively

Moreover, with the screening of a set of 95 R- and B-lines (Supplemental Table 4) for the most promising KASPs on chromosomes SBI-02 and SBI-05, the marker Sobic.002G057050_1090 discriminated R- and B-lines most accurately. This marker had a false positive rate of 0.0% (45 out of 45 correct) for B-lines and 26.0% (37 out of 50 correct) for R-lines. All the other KASPs had much higher false positive rates (Supplemental Table S3).

## Discussion

This study is the first known on the genetics of fertility restoration in the A_1_ cytoplasm for West African breeding materials. In addition, this is the first known use of Guinea-race derived materials/germplasm for such study. CK60A, one of the two female parents, is a well-known male-sterile female line used in breeding programs in WA and worldwide. FambeA, DT_298 and Lata are derived from local germplasm and used in local breeding programs, thus allowing a very suitable basis for mapping fertility restorer genes in the WA sorghum germplasm. Markers developed from this study would be a valuable tool for direct use in WA sorghum breeding programs.

### Phenotyping and genetics of fertility restoration in WA germplasm

The patterns of fertility score distributions showed consistency that suggests that the scoring was effective for phenotypic evaluation of fertility restoration. Parents showed no variation from the expected phenotypes. POP_FL_ followed the anticipated distribution having more fertile than sterile plants, but also a continuum of partially restored fertility, as in the two other F_2_ populations. A continuum of partially restored fertility, showing the presence of partial restorer genes, was likewise found in sorghum fertility restoration studies (Jordan et al. 2010, 2011). The distribution of fertility score in POP_CD_ and POP_FD_ was contrary to what is generally reported in sorghum (Klein et al. 2001; Jordan et al. 2011; Praveen et al. 2015, 2018), with most of plants being sterile (Fig. 1). The fertility score distributions of the “repeated” F_2_ populations were similar to the corresponding F_2_ set used for the mapping study (data not shown). Extreme temperatures (mostly low night temperatures) around heading date could reduce the pollen quantity and viability, and consequently the seed set, in sorghum (Downes and Marshall 1971; Brooking 1976; Prasad et al. 2006; Hatfield and Prueger 2015). Therefore, we looked at the temperatures around heading time during our experiments and could not find any influence of temperature on fertility score; neither had we found significant correlations between heading date and the fertility score in the F_2_ and F_3_ populations (Fig. 1, Supplemental Table S4). Hence, we concluded that no specific year effects, e.g., extreme weather events, confounded our fertility reaction phenotyping.

Consequently, distributions in the mapping populations, as well as those of the validation sets and the *χ*^2^ tests, indicate that, as previously described in sorghum (Maunder and Pickett 1959; Miller and Pickett 1964; Wen et al. 2002; Jordan et al. 2010, 2011), one or multiple dominant loci plus modifier and/or partial restorer genes may control the fertility restoration in WA sorghum. One single gene seems to control the fertility restoration/sterility maintenance in POP_FL_, whereas the genetics of fertility restoration/sterility maintenance in POP_CD_ and POP_FD_ are not well understood with more sterile than fertile plants; hence, further studies would be needed for clarification. However, the low phenotypic variance explained by our markers on chromosome SBI-05 in the POP_CD__F_3_ validation population, as well as the phenotypic distributions of POP_CD_ and POP_FD_, points to a highly quantitative trait with multiple potential partial restorer genes.

### Fertility restoration loci and partial restorers

The removal of distorted sites prior to the genetic map construction provided us with a less dense but more accurate linkage map, by avoiding bias in marker order and in the distances between distorted markers (Lorieux et al. 1995; Liu et al. 2010). Distorted markers were reported in various mapping studies (Mace et al. 2009; Kong et al. 2018; Boyles et al. 2017) and were associated with the population type, the specific cross and the type of molecular marker. In our study, the difference in amount of distorted markers between populations suggests that the difference between parental lines may have influenced the segregation distortion (Paterson et al. 2009). Further, most of distorted markers (19, 15 and 23% in, respectively, POP_CD_, POP_FD_ and POP_FL_) were located and evenly distributed on chromosome SBI-01. This chromosome was reported to contain most of the distorted makers in various sorghum mapping studies (Menz et al. 2002; Mace et al. 2009; Kong et al. 2018). However, for a higher coverage, inclusion of these markers in the linkage map construction should be considered with more fitting statistical models (Lorieux et al. 1995; Xu 2008). A deeper characterization and insight of the excluded distorted sites would be worthwhile for breeding purposes, given that the population with 100% Guinea-race background presented a higher rate of distorted markers and distortion favored generally the male parent’s alleles.

The QTL region on chromosome SBI-05 found in POP_CD_ and POP_FD_ is located in the same 2.6-Mbp region detected by Jordan et al. (2011) as a major locus for fertility restoration and described as *Rf*_5_ locus, using Australian material. This major locus restored fertility in both A_1_ and A_2_ cytoplasms and was associated with a modifier or partial fertility restorer locus on chromosome SBI-04. We found a small effect QTL on the same chromosome SBI-04 in POP_CD_ that was different from the one found in the above-cited study. It is possible that major loci are common across Australian and West African germplasm, and, depending on environmental conditions, the partial restorer loci will be expressed or their effects masked in a particular genotype. With more phenotypic variance explained, QTL on chromosomes SBI-05 in POP_CD_ and POP_FD_ had relatively larger effects than those on chromosomes SBI-02, SBI-03 and SBI-04. The effect of the minor QTL on chromosome SBI-10 in POP_FD_ was large and rather similar to the effect of the major QTL on chromosome SBI-05 (Fig. 2, Table 3). The small sample size of POP_FD_ could have led to the likely overestimation of its effect (Utz and Melchinger 1994), whereas QTL with larger effect on chromosome SBI-05 could still be detected (Vales et al. 2005). Further, the fact that we found the same QTL region on SBI-05 for POP_CD_ and POP_FD_ (same male parent) gives more confidence in this major QTL than on the population-specific minor QTL. Small effect QTL need therefore more focus with larger and repeatable populations in order to more accurately map them, estimate their true effects, and understand how they affect fertility restoration in WA germplasm in different environments.

The effect of the only QTL found on chromosome SBI-02 in POP_FL_ was largest across populations with 31% of the variance explained (Table 2). This indicates that despite the relative low marker density in that region (Fig. 3) and the relatively limited population size, this QTL stands as a strong candidate for fertility restoration. However, the power and the accuracy of detected QTL could be decreased as compared to larger populations (Schön et al. 2004). Further, that same region had been mapped and described as *Rf*_2_ locus, restoring male fertility in the A_1_-type CMS in Australian germplasm (Jordan et al. 2010), and recently in Indian germplasm (Praveen et al. 2018). This locus seems therefore to control fertility restoration in some WA lines, as well as in the Australian and Indian germplasm.

Further, Caudatum, Kafir, Durra and inter-racial lines (Additional Table 1 in Mace et al. (2008)) were used in the studies of both Jordan et al. (2010) and Praveen et al. (2018), while our POP_FL_ has a 100% Guinea-race background. Thus, there is no evidence that the fertility restoration loci are race specific. Identified QTL or developed markers should therefore be useful either for Guinea-race-oriented breeding programs or for the development of non-Guinea-race hybrid parents.

### PPR-*Rf* genes and a potential causative mutation for *Rf*_2_

The capacity of *Rf* genes to encode mitochondria-targeted PPR proteins was first discovered by the cloning of *Rf*-*PPR592* gene from petunia (Bentolila et al. 2002). Further, except the *Rf*_2_ gene in maize, which is a member of the aldehyde dehydrogenase gene family (Cui et al. 1996; Liu et al. 2001), all *Rf* genes identified so far in other crop species, including rice (Kazama and Toriyama 2003; Komori et al. 2004; Inagaki et al. 2004) and sorghum (Klein et al. 2005), are members of the PPR gene family. Rf-like PPR genes evolve rapidly (Dahan and Mireau 2013) through a “birth and death” process (Geddy et al. 2007), and their local duplication occurs rather frequently, for them to ensure corrective functions (Dahan and Mireau 2013). In our study, clusters of PPR genes were found in the QTL regions. Since we could only use gene model data of the currently available reference genome, it is possible that in our tested parental lines further PPR gene duplicates are present. In POP_CD_, POP_FD_ and POP_FL_, we found 12, 13 and 7 PPR genes, respectively, spanning 2.01, 1.30 and 1.87 Mbp genomic segments (with a respective average distance of 179, 257 and 152 kbp between PPR genes), on the QTL regions on chromosome SBI-05 (POP_CD_ and POP_FD_) and chromosome SBI-02 (POP_FL_). We could not sequence all PPR genes in these regions and therefore only selected the ones located in—or linked to—the confidence intervals for validation and marker development.

In the region spanning the *Rf*_5_ locus, Jordan et al. (2011) identified a cluster of six PPR genes exhibiting a strong homology with the rice *Rf*_1_ gene. The four shared PPR genes to POP_CD_ and POP_FD_ were located in a region of sorghum chromosome SBI-05 from 0.98 to 2.37 Mbp (Table 2), whereas in the above-cited study, the six candidate PPR genes for the *Rf*_5_ locus were located in the region from 2.45 to 2.78 Mbp of the chromosome SBI-05. Therefore, there was no overlap of PPR genes between these two studies. In the present study, we could not detect any plausible mutations in any of the sequenced PPR genes on chromosome SBI-05. However, due to our reduced set of sequenced genotypes and sample size, we cannot rule out any direct impact of the sequenced genes, and hence, further research is needed here.

On chromosome SBI-02, we sequenced several PPR genes including Sobic.002G057050 (Sb02g004810 in the previous version of sorghum reference genome) which was proposed by Jordan et al. (2010) as a candidate gene for fertility restoration in the A_1_ CMS system. Jordan et al. (2010) suggested that additional fine mapping, along with additional experimental observations, would be necessary to confirm the identity of the *Rf*_2_ gene. Very recently, the 236 kb *Rf*_2_ locus was fine mapped to 10.32 kb and Sobic.002G057050 was the only PPR gene located in this interval (Praveen et al. 2018). The same study found that Sobic.002G057050 was more expressed in the sorghum inflorescence as compared to the stem or the leaves, and 12 times more expressed in inflorescence tissues of male-fertile (restorer) lines as compared to the male-sterile (female) ones. Further, Praveen et al. (2018) found 44 and 43 SNPs between the parental female line (296A) and the two restorer lines (RS29 and DSV1, respectively) used in their study, and a 3 bp deletion in the coding DNA sequence of 296A. In our study, we found the same deletion in all the sequenced B-lines and in one of our R-lines as well (Fig. 4). Further, we detected a missense mutation (1090 bp) leading to an amino acid exchange and a clear separation of B- and R-lines. This same SNP was also found by Praveen et al. (2018) separating their A- and R-lines (Supplemental Fig. S2). The developed marker from this mutation, namely Sobic.002G057050_1090, explained around 81% of the phenotypic variation in our POP_FL__F_3_ validation population (Table 4). All genotyped B-lines were carrying the recessive (female/maintainer) allele of Sobic.002G057050_1090, and 37 out of the 50 genotyped R-lines had the male parent’s allele (Supplemental Table S3). The few R-lines that did not carry the restorer allele of Sobic.002G057050_1090 (excluding the heterozygous status) had the restorer allele for marker S5_1180493 on chromosome SBI-05. This points to the two major loci *Rf*_2_ and *Rf*_5_ as being complementary; hence, either of them must be present to restore fertility in the tested material. We must point out though that most of the characterized B-lines had the same or similar genetic background. For a better characterization of WA B-lines, genotypes with more diverse background have to be assessed with the developed markers. Putting the results of Jordan et al. (2010), Praveen et al. (2018) and ours together point very strongly to Sobic.002G057050 as the underlying gene of the *Rf*_2_ locus and to the SNP at 1090 bp as the causative mutation. Awaiting the validation of Sobic.002G057050, Sobic.002G057050_1090 can be a useful tool for an initial molecular identification and selection of maintainer and restorer lines in WA breeding programs; nonetheless, caution must be paid to the partial restorers and the QTL on chromosome SBI-05.

### Markers and options for increasing effectiveness of selection for hybrid parents in WA

Maintaining the sterility of A-lines while duplicating their seeds is necessary for commercial hybrid seed production. Maintainers should not have restorer alleles to prevent male fertility restoration of female lines while maintaining A/B pairs. Apart from the already-discussed locus on chromosome SBI-10 of POP_FD_, QTL with smaller effects poorly explained the phenotypic variation in the respective mapping populations (Fig. 2, Table 3). In any case, these QTL regions may contain partial restorer genes and understanding how they affect the fertility restoration is essential for breeding programs. A-lines must possess the highest level of male sterility to ensure 100% hybrid seed production. Partially fertile A-lines lead to a degradation of hybrid seed quality and can reduce hybrid purity and yield in field crops. Markers for fertility restoration/sterility maintenance, if available, would allow a marker-assisted assessment of fertility restoration or sterility maintenance of WA breeding lines, and could be more efficient, labor- and cost-effective than direct field evaluation. Such markers would help excluding the (partial) restorer alleles from the A- and B-lines, as partial fertility restorer genes can stay unobserved for generations and be expressed under particular environmental conditions (Jordan et al. 2010, 2011). Alternatively, the combination of major restorer genes/loci and partial restorers can lead to a recovery of complete fertility in the F_1_ hybrids. Therefore, understanding and introgressing several *Rf* loci, either full or partial restorer, in restorer lines used in the breeding programs could lead to complete restoration of fertility in hybrids and therefore ensure a more complete panicle filling for more grain yield. However, until molecular tools will be at hand, including for minor QTL found in this study, breeders would still need to rely on extensive multi-location phenotypic evaluations to identify stable R- and B-lines in the advanced generations, specifically in environment corresponding to seed production.

## Conclusion

The final markers created in this study are the first set of markers for fertility restoration in WA sorghum germplasm. QTL on chromosome SBI-05 and partial restorers need to be better understood. The marker developed from the mutation in 1090 bp of the PPR gene Sobic.002G057050 clearly separated B- from R-lines. This, along with the high phenotypic variance the marker Sobic.002G057050_1090 explained in the F_3_ population and previous researches confirm that this gene is a strong candidate for fertility restoration in the WA A_1_ CMS, and that the mutation in 1090bp may be the causative mutation underlying *Rf*_2_. QTL on chromosomes SBI-02 and SBI-05 seem to work separately. Therefore, either combining both QTL in- or excluding them from- one genotype to ensure more stable R- or A/B-lines could be beneficial for WA breeding programs. For a certain set of genotypes, WA breeders can directly use Sobic.002G057050_1090 to preselect potential B- and R-lines. Nevertheless, and awaiting for the validation of PPR Sobic.002G057050, this marker and its stability would need to be assessed on genetically more diverse B-lines and more environments.

### Author Contribution statement

HFR, WLL, EW and BIGH conceived the study. MK, BN and WLL collected the data; MK performed the analyses; MK, WLL, BIGH, and HFR wrote the paper. All authors approved the final manuscript.

## Electronic supplementary material

Below is the link to the electronic supplementary material.

Supplementary material 1 (DOCX 1217 kb)

## Abbreviations

CMS: Cytoplasmic male sterility
CTAB: Cetyltrimethylammonium bromide
DNA: Deoxyribonucleic acid
GBS: Genotyping by sequencing
ICRISAT: International Crops Research Institute for the Semi-arid Tropics
IER: Institut d’Economie Rurale
KASP: Kompetitive allele-specific polymerase chain reaction
ORF: Open reading frame
PCR: Polymerase chain reaction
PPR: Pentatricopeptide repeat
QTL: Quantitative trait loci
Rf: Fertility restoration locus/gene
SNP: Single nucleotide polymorphism
WA: West Africa
