# Supplementary material for: QTL mapping and validation of fertility restoration in West African sorghum A1 cytoplasm and identification of a potential causative mutation for Rf2
Source: Theor Appl Genet. 2018 Aug 21;131(11):2397–412. doi: 10.1007/s00122-018-3161-z (PMC6208960; doi:10.1007/s00122-018-3161-z)
Supplement: Supplementary file 1 — Supplementary material 1 (DOCX 1217 kb) [file 122_2018_3161_MOESM1_ESM.docx]

**Supplemental files**


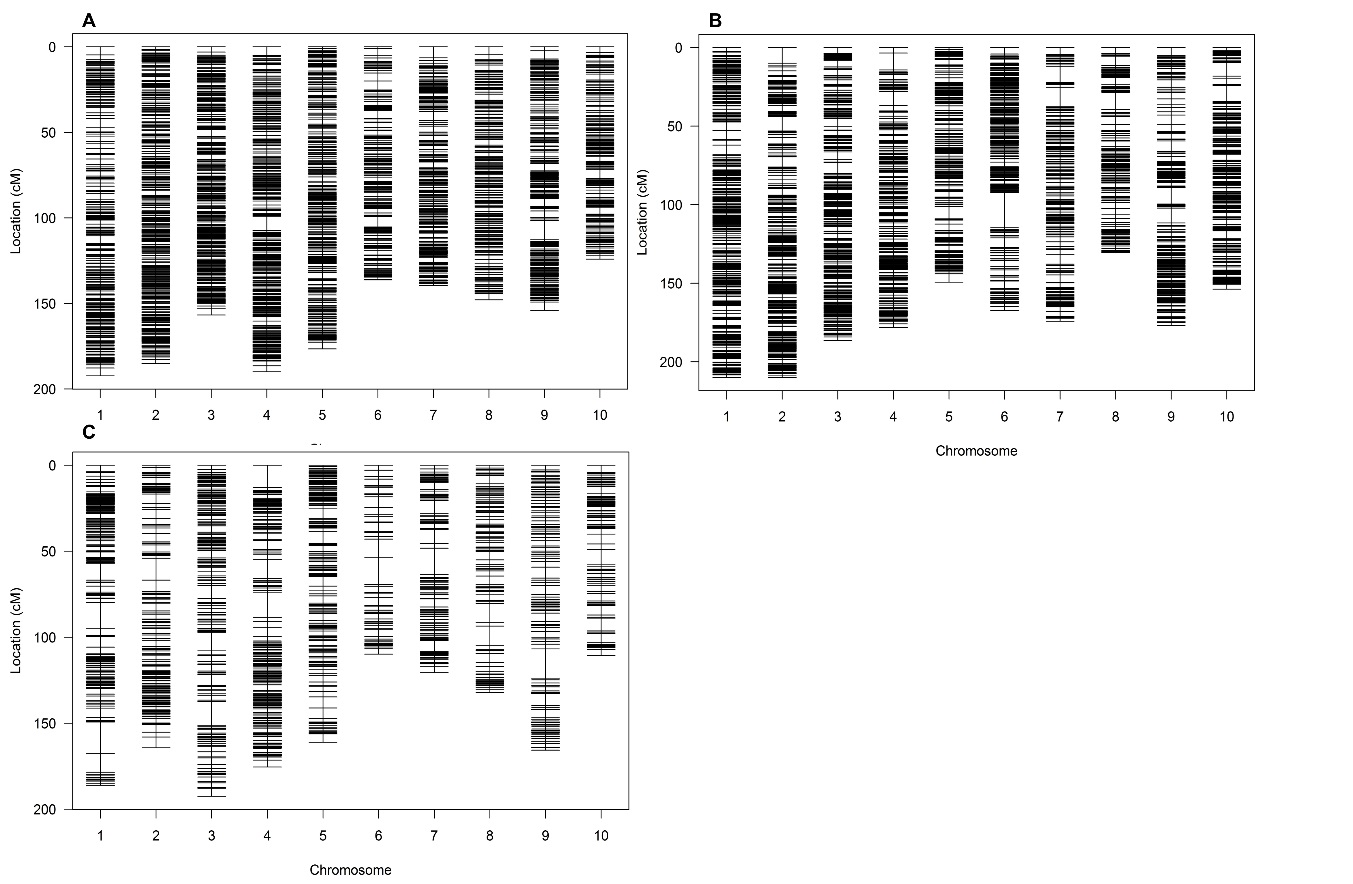


**Supplemental Fig. S1** Genetic linkage maps from three sorghum F_2_ populations. POP_CD_ (A) and POP_FD_ (B) are from the crosses between male DT_298 and respectively CK60A and FambeA as female parents. POP_FL_ (C) is from the cross between Lata and FambeA


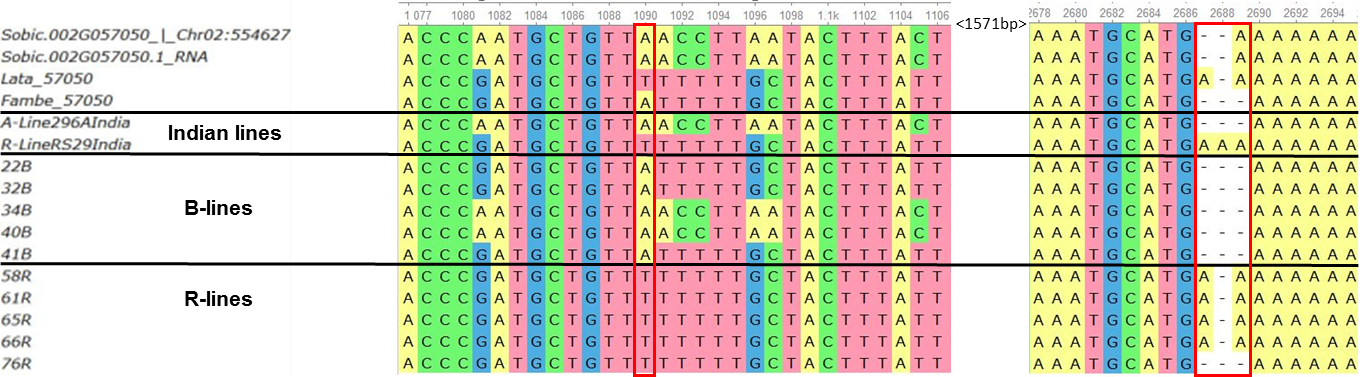


**Supplemental Fig. S2** Aligned sequences of Sobic.002G057050 around the two potential causative mutations at 1090bp and 2686bp. Two first rows show the reference genome, followed by the parents of POP_FL_ mapping population, Lata (R-line) and Fambe (A-line), followed by two sequenced Indian lines (A-line 296A and R-line RS29) (Praveen et al. 2018), followed by five B-lines and five R-lines sampled from ICRISAT breeding material (Supplemental Table S3)

**Supplemental Table S1** SNPs, flanking DNA sequences, primer sequences and polymerase chain reaction (PCR) conditions used in the KASP marker development

| **Family** | **Cross** | **KASP-ID** | **Position in cM** | **Chr** | **Position in bp** | **PCR Condition** | **Sequence around SNP** | **FAM** | **HEX** | **Com** | **With FAM Tail** | **With Hex Tail** |
| --- | --- | --- | --- | --- | --- | --- | --- | --- | --- | --- | --- | --- |
| POP_FL_ | FambeAxLata | S2_6045380 | 22.19 | 2 | 6045380 | 61-55°C | AATCTTATGATCTTTGTTCGAGACACTTCCAAAGTCTCCAAATACTGCAGCCTGGCTACCTCTGTTGGGATCTCAGTAATCCCCTCTCCACCAGACACTA**[G/A]**CAGGTGCCTGAGACGGTGCAGCCCACACATATCCTTCAGATGTTGATTCTCGAGAACCCCACTGTTGTTTCGAGCGTCCAACACTCGCAGCTGTTGCAAATGCTTGTAAGGGATCTTCTCAGCATCTTCTAAAACGACAAGGGACCGGACATGATGCCAATCATCAATTCCCGACAAGGGATCCACTGGATCACTGCTTG | ATCCCCTCTCCACCAGACACTAG | ATCCCCTCTCCACCAGACACTAA | GATGCTGAGAAGATCCCTTACAAGC | GAAGGTGACCAAGTTCATGCTATCCCCTCTCCACCAGACACTAG | GAAGGTCGGAGTCAACGGATTATCCCCTCTCCACCAGACACTAA |
| POP_FL_ | FambeAxLata | S2_6843380 | 24.55 | 2 | 6843380 | 61-55°C | CCTACCAACCAATCACTTCTCAGTTTTATTCTTACAGAAACTAAGGCTCGAGTAGAGAGACGCGGCTGCTGTCCAGGAGGTGACATTTCCACCTCGTCCA**[A/G]**GAGCTTTCCAGGAGCACGGATGCCGTCTAGGAGGTGACTAGCTGCAGCACAGCTACTGTCCAGGAGCATGGCTAGCTGCAGCAATTTTATACAAATTTCA | GTGACATTTCCACCTCGTCCAG | GTGACATTTCCACCTCGTCCAA | TGTGCTGCAGCTAGTCACCTCCTAG | GAAGGTGACCAAGTTCATGCTGTGACATTTCCACCTCGTCCAG | GAAGGTCGGAGTCAACGGATTGTGACATTTCCACCTCGTCCAA |
| POP_FL_ | FambeAxLata | S2_7406429 | 25.82 | 2 | 7406429 | 61-55°C | TCATTTGAGGTACCGCACACAAGGCCTGACTTGGGTATGTGAACAGATTTATGAAGGAGCCCCATGAGGAGCATCTGGCAGCAGTGAAACATAATTTGAG**[G/C]**TTCATCATAGCTGGGACTAGAAGCTGGGGTCTCTTATAGCCCAGGATGGAGGAAGGTGCAGCACTACATGGGTTGAGTGATAGTCATCTTGGAGGGGACA | GGCAGCAGTGAAACATAATTTGAGG | GGCAGCAGTGAAACATAATTTGAGC | CAACCCATGTAGTGCTGCACCT | GAAGGTGACCAAGTTCATGCTGGCAGCAGTGAAACATAATTTGAGG | GAAGGTCGGAGTCAACGGATTGGCAGCAGTGAAACATAATTTGAGC |
| POP_FL_ | FambeAxLata | S2_8715821 | 30.90 | 2 | 8715821 | 61-55°C | TGTGTTTCTGGTCCGTACACCGGAGGAACAAGAAGCCTGGCAACGGCAACAAGCCCTGCTGCGATAAGATCCCGGTGTCCCAAATCCCGGACGTCTCCAA**[A/G]**GAGATTGCGGTGGACGAGGTGCGGGAGCAGCACCACGCCGTTGTCCACAACCTCCGTGTGCAGCAGGAAAGCCATCACACGCTGGCCGTTCAGGAGAAGC | CAAATCCCGGACGTCTCCAAG | CAAATCCCGGACGTCTCCAAA | CTGCACACGGAGGTTGTGGAC | GAAGGTGACCAAGTTCATGCTCAAATCCCGGACGTCTCCAAG | GAAGGTCGGAGTCAACGGATTCAAATCCCGGACGTCTCCAAA |
| POP_CD_ + POP_FD_ | CK60AxDT_298 + FambeAxDT_298 | S5_1610046 | 10.33 | 5 | 1610046 | 65-57°C | GGCGGGTGTAAAAATGGCACCTTTGGGTGTCGCTCTGCTGCTGATGCTGCTGGAGCAGGAAGCTGAGGCTGACGCCAGCTCCTTTGGGCGACGGCGAGAA**[C/A]**GGGTACGCGGACGCCATGAGCTGCGGCGGCGGCGCGGCGGCGAGGGCCTTGGCGCGCATGACGGCCATCTCCCGCTCCGCGGCGTCGTCCTCAGGCACGCCGTGGAGGAAGCGGCAGGAGGAGCCGTTCTTGCAGTACCCGCGCGCGTAGTACATGCAGGGCCTCCACGACGCGGCCGCCGCCTCGGCGTCGCTGAGCGA | TGGGCGACGGCGAGAA**A** | TGGGCGACGGCGAGAA**C** | GCCGCAGCTCATGGCGT | GAAGGTGACCAAGTTCATGCTTGGGCGACGGCGAGAAA | GAAGGTCGGAGTCAACGGATTTGGGCGACGGCGAGAAC |
| POP_CD_ + POP_FD_ | CK60AxDT_298 + FambeAxDT_298 | S5_2547529 | 20.52 | 5 | 2547529 | 61-55°C | CGGGCGCCGCCGTCCAGTAATCCGCGTCGAAGTGGTGTTCCTCCTGCTGGCGCGGCGGCACGCGCTCCCGCTGCCTCTTGTTGCACGGGGCCGGCACGTC**[G/C]**TTGGCCTCGAGCTGCTGCTGGAGCTCGTCGCCGTCCGCCGCCGCCGCCGCCACCGCCACCGTCGCCTTGGGTCTGAGGTGCAGCCTGCAGAGCACCCTGA | CAGCAGCTCGAGGCCAAC | CAGCAGCTCGAGGCCAAG | AAGTGGTGTTCCTCCTGCTGG | GAAGGTGACCAAGTTCATGCTCAGCAGCTCGAGGCCAAC | GAAGGTCGGAGTCAACGGATTCAGCAGCTCGAGGCCAAG |
| POP_CD_ + POP_FD_ | CK60AxDT_298 + FambeAxDT_298 | S5_3626674 | 24.08 | 5 | 3626674 | 61-55°C | TTGCTGCCAGTACTGCTGCTCTCGCCGTCGTCGAGCAACTCCGCCTGCGGCGGGCAGCGCTTCTTGTGCACGTCCCAGCGCTCGCCGGATGATGCGCGGC**[T/C]**GATGGGAGACGTGCCTGAACATGACGACGACGACGAAGCTGGGCTTGCTGCCTTCTTGTGCGCGTCCCACCTCTCAGCGGAGTCGGCCCGGCCGGGCTTC | CGGATGATGCGCGGCC | CGGATGATGCGCGGCT | CGCACAAGAAGGCAGCAAG | GAAGGTGACCAAGTTCATGCTCGGATGATGCGCGGCC | GAAGGTCGGAGTCAACGGATTCGGATGATGCGCGGCT |

**Supplemental Table S1** Continued

| **Family** | **Cross** | **KASP-ID** | **Position in cM** | **Chr** | **Position in bp** | **PCR Condition** | **Sequence around SNP** | **FAM** | **HEX** | **Com** | **With FAM Tail** | **With Hex Tail** |
| --- | --- | --- | --- | --- | --- | --- | --- | --- | --- | --- | --- | --- |
| POP_CD_ | CK60AxDT_298 | S5_1608322 | 10.33 | 5 | 1608322 | 65-59°C | CAAGCAGCATTGGGTGTTGCTGTCACAGGACCATATTCCACCTTCTCCTCTTCCCCAGGGTTGACTGCCTGCTTCTGCTTGGGGCTGCCATCACCACCCTCTCCCTGCTGCTCCTCGTGCTTGCCTTCTGCATCAGCAGTTGCAGAGGCAGCAGCAGGAGCACTCATTGCAACGCTACTAGTACTGCTCATCCTCTCTGC**[G/A]**ATCAGAAAAAAGCATGGATTCAAAAAGTCACAACCTTGCCAACACCTCACCAAAATGCTGCAATGCAAGAGGCAAGAACTTGTGAGGGTCAAGGAGTGGACCTTGGATGCCGGTGACATCCTCCAAATGGGAGGCATTGCCGCCATTCCCGTGGTTGCCGCTGCCTTTGCCATCAGCTTGCCCCAGAGACATGGGCGAAC | TAGTACTGCTCATCCTCTCTGCG | ACTAGTACTGCTCATCCTCTCTGCA | ACTCCTTGACCCTCACAAGTTCTT | GAAGGTGACCAAGTTCATGCTTAGTACTGCTCATCCTCTCTGCG | GAAGGTCGGAGTCAACGGATTACTAGTACTGCTCATCCTCTCTGCA |
| POP_CD_ | CK60AxDT_298 | S5_1180493 | 4.84 | 5 | 1180493 | 65-59°C | aataatttataaaataaattttttaaatttaattagtttataattatacagtagatattattaaataaaacgaaaatactgccgcacctgttgaaacacctccaaaaacaGGCCGGACGTCTCCTAGTCCTCTGCTACCTCCATGGCCGGAAgccgccgccgccgccgccgccgcAGAGCGCGATCGTCGCAGCAGAGCG**[T/C]**CGGATCCTCGTCGCTGGAGCCGGAGGGCGCCACTCTCAGCACCAGTCTGCAGCCTGACCGCAACCCTAACCGCTCATCTCTGTTCCTTCCCCCTCGCTTCTTACTTTAGGGCGTATTTATTTCCTGAGCGCATGGAGGCGGATCCAAGACAGAAATAGGAGAGAATCCGCGAGTTGTTGCAACCAAAAATCAATCAGTAG | GATCGTCGCAGCAGAGCGT | ATCGTCGCAGCAGAGCGC | CCTAAAGTAAGAAGCGAGGGGGAAGG | GAAGGTGACCAAGTTCATGCTGATCGTCGCAGCAGAGCGT | GAAGGTCGGAGTCAACGGATTATCGTCGCAGCAGAGCGC |
| POP_FD_ | FambeAxDT_298 | S5_2174322 | 7.70 | 5 | 2174322 | 65-59°C | GTATGTCGTCTGAGATTGCCATGTAGTTGAACCTACACGCGTATATATTATTCATGCACAAGCTTGGTATTGGTCACAGGTGTAAAAGATAGCATCTTtatatatatatatatatatatatatatGTGCGATGAGAAATGTAAATATATATAGTGTTATATATATGATTGTTGTCTGAAGAACAATATGCAGCATTGCAT**[G/C]**GTTACTCTAGTACTGACATGTCTGCGTTGAGCTTGAAAGCCAGCCGTGCCTCCGAGACGTTGAGAGCAGGGCAGCTGCTATTGTGCTCGAAGATGGCGTAGCAGTAGAACCCGGAGCTGCCTTTCAGCATCACAAACCTAGAAAGGATCCAACAACAACAGTTAATTTCTTTAACTGTCACAGGTATGTAACTTGTGTGT | AGAACAATATGCAGCATTGCATC | AGAACAATATGCAGCATTGCATG | TTTCAAGCTCAACGCAGACAT | GAAGGTGACCAAGTTCATGCTAGAACAATATGCAGCATTGCATC | GAAGGTCGGAGTCAACGGATTAGAACAATATGCAGCATTGCATG |
| POP_FL_ | FambeAxLata | S002G057050_1090 |  | 2 | 5547362 | 65-59°C | TGGGTATTTCATAGCTGGTGAAGTGGGCGAAGTGATGAAGCTCCTTGATGAGATGCTCTTGATTGGCTTGAAACCCGATGCTGTT**[T/A]**TTTTTGCTACTTTATTTGATGGCATGGTCTCTAAGGGATTGAATCCTGATGTTGACACATGTAAGACTTTGATTGATAGCTGCTGTGAAGATGACAGGATAGAGGATATATTAACTCTGTTCCGAGAAATGTTGAGCAAGGCTGATAAGACTGACACTATCACGGAAAATATAAAACTGTGAGTGTCACTTCAGAATCGACGGACTGCCATTGGGATGGAACTCAAGCTGCAGATGGCCAAAAGGGTTCAA | GCTTGAAACCCGATGCTGTTA | GCTTGAAACCCGATGCTGTTT | CCTGTCATCTTCACAGCAGCTATC | GAAGGTGACCAAGTTCATGCTGCTTGAAACCCGATGCTGTTA | GAAGGTCGGAGTCAACGGATTGCTTGAAACCCGATGCTGTTT |

**Supplemental Table S2** Primer sequences and PCR conditions used for the Sanger sequencing of several PPR genes

| **Gene** | **Primer Name** | **Primer Use** | **Sequence (5'-3')** | **Annealing Temp** | **Taq** |
| --- | --- | --- | --- | --- | --- |
| Sobic.002G054100 | Sobic.002G054100_F | PCR/Sequencing | TTTCGGATCGCGGACAGAAA | 51°C | New England Biolabs Phusion/Q5 |
| Sobic.002G054100 | Sobic.002G054100_R | PCR/Sequencing | GCCTTGCCCAACATTTCTCG |  |  |
| Sobic.002G057050 | Sobic.002G057050_F | PCR/Sequencing | CTTCGGCATCCTCATCAGCT | 68°C | New England Biolabs Phusion/Q5 |
| Sobic.002G057050 | Sobic.002G057050_R | PCR/Sequencing | CTCCAGACTGATGCACATCCA |  |  |
| Sobic.002G059700 | Sobic.002G059700_F | PCR/Sequencing | AGCTTCCATTCGTTCCCAGG | 62°C | New England Biolabs Phusion/Q5 |
| Sobic.002G059700 | Sobic.002G059700_R | PCR/Sequencing | AGCGCAACATAACACGTCCT |  |  |
| Sobic.005G011000 | Sobic.005G011000_1_R | PCR/Sequencing | TAGTGCCAGTGCTAGAGCTG |  |  |
| Sobic.005G011000 | Sobic.005G011000_F | PCR/Sequencing | TTGGGGCGAACTAAACGAGG | 62°C | New England Biolabs Phusion/Q5 |
| Sobic.005G017100 | Sobic.005G017100_1_F | PCR/Sequencing | ATACGGCCCAACAACCCAAT | 68°C | New England Biolabs Phusion/Q5 |
| Sobic.005G017100 | Sobic.005G017100_1_R | PCR/Sequencing | ACTCATGCATGTCTCGTGAACA |  |  |
| Sobic.005G020600 | Sobic.005G020600_1_F | PCR/Sequencing | AAATCGAATGCGGGATCGGA | 67°C | New England Biolabs Phusion/Q5 |
| Sobic.005G020600 | Sobic.005G020600_1_R | PCR/Sequencing | CGGCTGTTCTTCCATGTCCT |  |  |
| Sobic.005G026400 | Sobic.005G026400_F | PCR/Sequencing | CCACCACCACTGCCATGG | 63°C | New England Biolabs Phusion/Q5 |
| Sobic.005G026400 | Sobic.005G026400_R | PCR/Sequencing | AGACTCGAAACAGCTTGGCA |  |  |
| Sobic.002G054100 | Sobic.002G054100_1404_F | Sequencing | GAGGCAGTCCTTCAGCAGAT |  |  |
| Sobic.002G054100 | Sobic.002G054100_410_F | Sequencing | TCCAATCTCCTGCCTCAGTT |  |  |
| Sobic.002G054100 | Sobic.002G054100_914_F | Sequencing | CATAGCTACCTTCGGCATCC |  |  |
| Sobic.002G057050 | Sobic.002G057050_1378_F | Sequencing | GAGGCATTCCTCCTAATGTCA |  |  |
| Sobic.002G057050 | Sobic.002G057050_1845_F | Sequencing | TGGCCAAAAGGGTTCAATAG |  |  |

**Supplemental Table S2** Continued

| **Gene** | **Primer Name** | **Primer Use** | **Sequence (5'-3')** | **Annealing Temp** | **Taq** |
| --- | --- | --- | --- | --- | --- |
| Sobic.002G057050 | Sobic.002G057050_2352_F | Sequencing | AAGCAAGCCAGTGAAAGCAT |  |  |
| Sobic.002G057050 | Sobic.002G057050_2876_F | Sequencing | GCCGGCATTTCTTTCTTTCT |  |  |
| Sobic.002G057050 | Sobic.002G057050_3379_F | Sequencing | CCTTCCTGGGAGCTGGAG |  |  |
| Sobic.002G057050 | Sobic.002G057050_3859_F | Sequencing | CTCTTCTCGAGGGGCTCTGT |  |  |
| Sobic.002G057050 | Sobic.002G057050_4361_F | Sequencing | TGAAAAACCCAATGTCAGCA |  |  |
| Sobic.002G057050 | Sobic.002G057050_857_F | Sequencing | CAGGGGCCAAATGTTGTTAC |  |  |
| Sobic.002G059700 | Sobic.002G059700_1370_F | Sequencing | CCCGGATGTCTTCTCCTACA |  |  |
| Sobic.002G059700 | Sobic.002G059700_1866_F | Sequencing | TTACCGAGGCCAAAACACTC |  |  |
| Sobic.002G059700 | Sobic.002G059700_338_F | Sequencing | GTCGATCCAAATGCGTTCC |  |  |
| Sobic.002G059700 | Sobic.002G059700_859_F | Sequencing | CCCGGATGTCTTCTCCTACA |  |  |
| Sobic.005G011000 | Sobic.005G011000_1257F | Sequencing | AAGGGAAAATGCACAGAAGC |  |  |
| Sobic.005G011000 | Sobic.005G011000_1736F | Sequencing | TCCCAATGTCGTGTTCTTCA |  |  |
| Sobic.005G011000 | Sobic.005G011000_2235F | Sequencing | TTCAGAGCCTGTGTTCTAAGGA |  |  |
| Sobic.005G011000 | Sobic.005G011000_651F | Sequencing | ATCCTTAAGACGGGCTGGAG |  |  |
| Sobic.005G017100 | Sobic.005G017100_1_1226F | Sequencing | AGAGCGGCGAGATGTGAG |  |  |
| Sobic.005G017100 | Sobic.005G017100_1_1703F | Sequencing | GCTTGTCGACTGATGTACAGG |  |  |
| Sobic.005G017100 | Sobic.005G017100_1_2222F | Sequencing | GGTAACCCCTTGTTGCTTCTC |  |  |
| Sobic.005G017100 | Sobic.005G017100_1_2713F | Sequencing | AACCAGAACAACATTTATGAAGCA |  |  |
| Sobic.005G017100 | Sobic.005G017100_1_674F | Sequencing | GTGGACGCCTACGTGAAGAG |  |  |
| Sobic.005G020600 | Sobic.005G020600_1_1248F | Sequencing | GCACATAGTGTACATTGCGTCA |  |  |
| Sobic.005G020600 | Sobic.005G020600_1_1297R | Sequencing | GTATTGCCGAGCGTTTTCAG |  |  |

**Supplemental Table S2** Continued

| **Gene** | **Primer Name** | **Primer Use** | **Sequence (5'-3')** | **Annealing Temp** | **Taq** |
| --- | --- | --- | --- | --- | --- |
| Sobic.005G020600 | Sobic.005G020600_1_1583F | Sequencing | TGAGAAGCACAGGAGGTTCA |  |  |
| Sobic.005G020600 | Sobic.005G020600_1_2570F | Sequencing | TATGCAGAGGCAACTCAGGA |  |  |
| Sobic.005G020600 | Sobic.005G020600_1_3074F | Sequencing | AGGCTAATGGCAGTGTCCAG |  |  |
| Sobic.005G020600 | Sobic.005G020600_1_3575F | Sequencing | CAGCTCCAGCTTCTGGTTTC |  |  |
| Sobic.005G020600 | Sobic.005G020600_1_553F | Sequencing | GCTCCTCTCGTCGGTAGTTG |  |  |
| Sobic.005G026400 | Sobic.005G026400_1411F | Sequencing | CAAACTCAGTGATGCGAGGA |  |  |
| Sobic.005G026400 | Sobic.005G026400_1909F | Sequencing | TTTGGTTAATGGGCTCACAA |  |  |
| Sobic.005G026400 | Sobic.005G026400_2408F | Sequencing | GCTCTTGGGATCAGGGTGTA |  |  |
| Sobic.005G026400 | Sobic.005G026400_2915F | Sequencing | TTCAGCTTCTCTTGCATAGCAC |  |  |
| Sobic.005G026400 | Sobic.005G026400_3409F | Sequencing | TCCATTGAGATGCGAAGTCA |  |  |
| Sobic.005G026400 | Sobic.005G026400_432F | Sequencing | CTGCTCAGGCTCCTCGTG |  |  |
| Sobic.005G026400 | Sobic.005G026400_903F | Sequencing | TGGCTGTTCTGAACCAGATG |  |  |

| **Pedigree** | | **Lab Id** | **Origin^b^** | **S2_6045380**^c^ | **S002G057050_1090^c^** | **S2_6843380^c^** | **S5_1180493**^d^ | **S5_2174322**^d^ |
| --- | --- | --- | --- | --- | --- | --- | --- | --- |
| Female/maintainer allele | |  |  | G | A | G | C | G |
| Restorer allele | |  |  | A | T | A | T | C |
|  | **B-lines^e^** | | | | | | | |
| **013-KO-F4DT-216-2 P3 CT** | | 38B | IER | A | A | A | C | G |
| **BCM-36-2** | | 37B | IER | A | A | A | C | G |
| **Fambe-F3-2 P2** | | 40B | ICRISAT | G | A | G | C | G |
| **Fambe-F3-4 P1** | | 41B | ICRISAT | G | A | G | C | G |
| **S1-1** | | 47B | Landrace | A | A | G | C | G |
| **S2-9** | | 48B | Landrace | G | A | G | C | G |
| **014-KE-ERT-321** | | 88R | IER | A | A | A | C | C |
| **014-SB-CS-F5-292** | | 90R | IER | G | A | A | C | G |
| (FambeB/PopD08_658)-2-1-9-6-4-4-9 | | 21B | ICRISAT | G | A | G | C | G |
| (FambeB/PopD08_658)-2-4-7-6-10-8-3 | | 23B | ICRISAT | G | A | G | T | C |
| (FambeB/PopD08_658)-2-4-7-6-2-5-5 | | 22B | ICRISAT |  | A |  |  |  |
| (POP/12B)-6-2-1-1-3-7-3-3 | | 14B | ICRISAT | A | A | A | C | G |
| (POP/12B)-10-1-2-6-6-6-19-4 | | 8B | ICRISAT | A | A | G | C | G |
| (POP/12B)-11-1-1-3-1-2-14-8 | | 9B | ICRISAT | A | A | A | C | G |
| (POP/12B)-11-1-2-13-6-9-18-6 | | 10B | ICRISAT | G | A | A | C | G |
| (POP/12B)-11-1-5-2-5-4-14-3 | | 11B | ICRISAT | A | A | A | C | G |
| (POP/12B)-11-1-7-1-5-5-9-4 | | 20B | ICRISAT | G | A |  | C | G |
| (POP/12B)-11-4-3-1-10-11-6 | | 29B | ICRISAT | A | A |  | C | G |
| (POP/12B)-11-4-3-1-5-3-5 | | 27B | ICRISAT | A | A | A | C | G |
| (POP/12B)-11-4-3-1-6-1-1 | | 28B | ICRISAT | G | A | A | C | G |
| (POP/12B)-11-5-2-9-5-9-6-11 | | 12B | ICRISAT | G | A | A | C | G |
| (POP/12B)-11-7-1-6-5-11-24-3 | | 13B | ICRISAT | G | A | A | C | G |
| (POP/12B)-1-3-3-1-3-11-22-9 | | 4B | ICRISAT | A | A | A | C | G |
| (POP/12B)-1-3-3-7-8-3-6-11 | | 19B | ICRISAT |  | A |  |  |  |
| (POP/12B)-2-3-3-13-7-5-6 | | 26B | ICRISAT | A | A | A | C | G |
| (POP/12B)-3-1-5-11-5-8-24-7 | | 5B | ICRISAT | G | A | A | C | G |
| (POP/12B)-3-1-7-2-8-7-1-7 | | 6B | ICRISAT | G | A | A | C | G |
| (POP/12B)-7-1-4-4-12-22-18-4 | | 7B | ICRISAT | G | A | A | C | G |
| (POP/12B)-7-4-3-1-10-6-6-10 | | 15B | ICRISAT | G | A | A | C | G |
| (POP/12B)-7-4-3-1-10-8-4-4 | | 16B | ICRISAT |  | A | A | C |  |
| (POP/12B)-7-4-3-1-4-11-2-2 | | 18B | ICRISAT |  | A | A | C | G |
| (POP/12B)-7-4-3-1-4-8-4-5 | | 17B | ICRISAT | G | A | A | C | G |
| (POP/PR3009B)-7-3-1-1-6-3-19-8 | | 1B | ICRISAT | A | A | A | C | G |
| (POP/PR3009B)-7-3-1-4-1-3-21-3 | | 2B | ICRISAT | A | A | A | C | G |
| (POPD08-622/12B)-8-3-3-10-10-1-6 | | 24B | ICRISAT | G | A | A | C |  |
| (POPD08-622/12B)-8-3-3-10-12-2-4 | | 25B | ICRISAT | A | A | A | C | G |
| 12B | | 32B | IER | G | A | A | C | G |
| 150B | | 33B | IER | G | A | A | C | G |
| CK60B | | 34B | ICRISAT | G | A | A | C | G |
| Dagli Kossurou | | 44B | Landrace | A | A | A | C | G |
| FambeB | | 30B | IER | G | A | G | C | G |
| GP271-20B | | 35B | IER | A | A | G | T | G |
| IPS0001B | | 36B | IER | G | A | G | C | G |
| PR3009B | | 31B | IER |  | A | A |  | G |
| ***B1-3***^f^ | | 43B | Landrace | A | A | A | C | G |
| **^a^** Genotypes in bold were tested on the field and characterized as R-lines, the fertility reaction of the other genotypes was already documented ^b^ ICRISAT: International Crops Research Institute for the Semi-Arid Tropics; IER: Institut d’Economie Rurale-Mali; Landrace from Malian sorghum collection ^c^, ^d^ Markers on chromosomes SBI-02 and SBI-05, respectively ^e^ POP= POPD08-611 and 12B= 02-SB-F5DT-12B ^f^ F_1_ hybrids of CK60AxB1-3 had mix panicles; therefore, B1-3 could not be characterized neither as a B- nor as an R-line. | | | | | | | | |

**Supplemental Table S3** Phenotypic^a^ and genotypic (color code and letters) assessment of the fertility restoration of several R- and B-lines from ICRISAT-Mali breeding material with newly developed KASP markers for QTL validation

**Supplemental Table S3** Continued

| **Pedigree** | | | **Lab Id** | **Origin^b^** | **S2_6045380^c^** | **S002G057050_1090^c^** | **S2_6843380^c^** | **S5_1180493^d^** | **S5_2174322^d^** |  |
| --- | --- | --- | --- | --- | --- | --- | --- | --- | --- | --- |
| Female/maintainer allele | | |  |  | **G** | **A** | **G** | **C** | **G** |  |
| Restorer allele | | |  |  | **A** | **T** | **A** | **T** | **C** |  |
|  |  | **R-Lines**^c^ | | | | | | | | |
| **015-SB-CS-DU-14** | | | 89R | IER | G | A/T | A | T | G |  |
| **015-SB-CS-DU-41** | | | 93R | IER | G | A/T | A | T | G |  |
| **016-SB-CS-DU-3** | | | 94R | IER | A | A | A | C | G |  |
| **04-CZ-F5P-52** | | | 87R | IER | G | T | A | T | G |  |
| **BCM-66-1** | | | 91R | IER | G | A | A/G | T | C |  |
| **BCM-70-2** | | | 85R | IER |  | A |  |  |  |  |
| **Fambe-F3-2 P1** | | | 39B | ICRISAT |  | A/T | A | C | G |  |
| **Filatimi** | | | 80R | ICRISAT | A | T | A | C | G |  |
| **Grinkan** | | | 77R | IER | G | T | A | C/T | C |  |
| **Koura** | | | 45B | Landrace | A | T | A | C | G |  |
| **Lata** | | | 78R | ICRISAT | A | T | A | C | G |  |
| **Lata//DouaG-2-4-1-1** | | | 65R | ICRISAT | A | T | A | C | G |  |
| **Lata//DouaG-5-18-1-1** | | | 67R | ICRISAT | A | T | G | C/T | C |  |
| **Lata//DouaG-6-6-1-1** | | | 68R | ICRISAT | A | T | A | C/T | C |  |
| **Lata//DouaG-6-8-1-1** | | | 69R | ICRISAT | A | T | A | T | C |  |
| **Lata//Grin-8-1-1-1** | | | 50R | ICRISAT | A | T | A |  |  |  |
| **Lata//Grin-8-2-1-1** | | | 51R | ICRISAT | G | T | A | C | G |  |
| **Lata//Grin-8-35-1-1** | | | 52R | ICRISAT | A | A/T | A |  | G |  |
| **Lata//Grin-8-39-1-1** | | | 53R | ICRISAT | A | T | A | T | C |  |
| **Lata//Grin-9-14-1-1** | | | 49R | ICRISAT | A | T | A | T | C |  |
| **Lata//Grin-9-4-1-1** | | | 54R | ICRISAT | A | T | A | T | C |  |
| **Lata//IS15401-6-19-1-1** | | | 55R | ICRISAT | A | T | A | C/T | C |  |
| **Lata//Ngol-1-27-1-1** | | | 61R | ICRISAT | A | T | A | C | C |  |
| **Lata//Ngol-4-7-1-1** | | | 64R | ICRISAT | A | T | A | T | C |  |
| **Lata//Ngol-7-17-1-1** | | | 63R | ICRISAT | A | T | A | T | C |  |
| **Lata//Ridb-8-9-1-1** | | | 73R | ICRISAT | A | T | A | C | G |  |
| **Lata//Samb-4-13-1-1** | | | 74R | ICRISAT | A | T | A | C | G |  |
| **Lata//Samb-5-2-1-1** | | | 75R | ICRISAT | A | T |  | C | G |  |
| **Lata//SC566-7-14-1-1** | | | 60R | ICRISAT |  | A |  | T | C |  |
| **M8-12** | | | 46R | Landrace | G | T | A | C | G |  |
| **Marakanio** | | | 82R | Landrace | G |  |  | T | G |  |
| **Seguifa** | | | 86R | IER | A | T | A | T | G |  |
| **Soubatimi** | | | 96R | ICRISAT | A | A/T |  | C | C/G |  |
| 02-SB-F4DT-298 | | | 84R | IER | A | A | A | T | C |  |
| 06-SB-F4DT-15 | | | 95R | IER | A | T | A | T | G |  |
| Diema | | | 81R | IER | A | T |  | C | G |  |
| Jiguiseme | | | 83R | IER | G | T | A | C | G |  |
| Lata//DouaG-4-2-1-1 | | | 66R | ICRISAT | A | T |  | T | C |  |
| Lata//DouaG-7-9-1-1 | | | 70R | ICRISAT | A | T | A | T | C |  |
| Lata//IS15401-6-32-1-1 | | | 56R | ICRISAT | A | T | A | T | C/G |  |
| Lata//IS15401-7-2-1-1 | | | 57R | ICRISAT | A | T | A | T | G |  |
| Lata//IS15401-8-10-1-1 | | | 58R | ICRISAT | A | T | A | T | C |  |
| Lata//Ngol-4-25-1-1 | | | 62R | ICRISAT | A | T | A | T | G |  |
| Lata//Ridb-3-9-1-1 | | | 71R | ICRISAT | A | T | A | C | G |  |
| Lata//Ridb-4-13-1-1 | | | 72R | ICRISAT | A | T | A | C/T | G |  |
| Lata//Samb-6-23-1-1 | | | 76R | ICRISAT | A | T | G | C | G |  |
| Lata//SC566-6-44-1-1 | | | 59R | ICRISAT | A | T | A | T | C |  |
| ***015-SB-CS-F5-219 P1 precoce guinea***^e^ | | | 42B | IER | A | A/T | A | T | G |  |
| ***08-SB-DU-149***^e^ | | | 92R | IER | G | T | G | T | C |  |
| ***Ngolofing***^e^ | | | 79R | Landrace | A | A | G | C | G |  |
| **^a^** Genotypes in bold were tested on the field and characterized as R-lines, the fertility reaction of the other genotypes was already documented ^b^ ICRISAT: International Crops Research Institute for the Semi-Arid Tropics; IER: Institut d’Economie Rurale-Mali; Landrace from Malian sorghum collection ^c^, ^d^ Markers on chromosomes SBI-02 and SBI-05, respectively ^e^ F_1_ hybrids had mix panicles; therefore, these lines could not be characterized neither as a B- nor as an R-line | | | | | | | | | |  |

**Supplemental Table S4** Correlation coefficient of phenotypic traits (pollen quantity, anther color, fertility restoration visual score, panicle weight, grain weight and seed number) recorded on POP_CD__F_3_ (upper triangle) and POP_FL__F_3_ (lower triangle) families used in the validation study

| Traits | Heading date | Pollen ^a^ | Color ^a^ | Score | Pa_W | Gr_W | Seed_Num |
| --- | --- | --- | --- | --- | --- | --- | --- |
| Heading date |  | -0.19 | 0.32^*^ | -0.11 | -0.28^*^ | -0.32^***^ | -0.31^***^ |
| Pollen | -0.14 |  | -0.33^***^ | 0.57^***^ | 0.50^***^ | 0.51^***^ | 0.49^***^ |
| Color | 0.53^***^ | -0.60^***^ |  | -0.12 | -0.23^*^ | -0.26^*^ | -0.26^*^ |
| Score | 0.01 | 0.78^***^ | -0.40^***^ |  | 0.64^***^ | 0.67^***^ | 0.68^***^ |
| Pa_W | -0.34^***^ | 0.69^***^ | -0.50^***^ | 0.72^***^ |  | 0.93^***^ | 0.93^***^ |
| Gr_W | -0.27^**^ | 0.68^***^ | -0.45^***^ | 0.70^***^ | 0.93^***^ |  | 0.98^***^ |
| Seed_num | -0.42^***^ | 0.69^***^ | -0.51^***^ | 0.74^***^ | 0.96^***^ | 0.97^***^ |  |
| ^*^, ^**^, ^***^: Significantly different from zero at α=0.5, 0.1 and 0.01. ^a^ The pollen quantity, the anther color and the fertility restoration score visual rating are detailed in the Materials and methods section. Pa_W, Gr_W and Seed_Num are the panicle weight, the grain weight and the seed number of individual panicles, respectively. | | | | | | | |
